# Interface Engineering of Ni_x_S_y_@MnO_x_H_y_ Nanorods to Efficiently Enhance Overall-Water-Splitting Activity and Stability

**DOI:** 10.1007/s40820-022-00860-2

**Published:** 2022-05-03

**Authors:** Pan Wang, Yuanzhi Luo, Gaixia Zhang, Zhangsen Chen, Hariprasad Ranganathan, Shuhui Sun, Zhicong Shi

**Affiliations:** 1 Institute of Batteries, School of Materials and Energy, Guangdong University of Technology, Guangzhou, 510006 People’s Republic of China; 2 Énergie Matériaux Télécommunications Research Centre, Institut National de La Recherche Scientifique (INRS), Varennes, Québec J3X 1P7 Canada; 3 The Key Laboratory of Fuel Cell Technology of Guangdong Province, School of Chemistry and Chemical Engineering, South China University of Technology, Guangzhou, 510641 People’s Republic of China

**Keywords:** Interface engineering, Protective shell, Manganese compound, Nickel sulfides, Bifunctional, Water splitting

## Abstract

**Highlights:**

- Three-dimensional (3D) core‐shell heterostructured Ni_x_S_y_@MnO_x_H_y_ nanorods grown on nickel foam (Ni_x_S_y_@MnO_x_H_y_/NF) were successfully fabricated via a simple hydrothermal reaction and a subsequent electrodeposition process.
- The fabricated Ni_x_S_y_@MnO_x_H_y_/NF shows outstanding bifunctional activity and stability for hydrogen evolution reaction and oxygen evolution reaction, as well as overall‐water‐splitting performance.
- The main origins are the interface engineering of Ni_x_S_y_@MnO_x_H_y_, the shell‐protection characteristic of MnO_x_H_y_, and the 3D open nanorod structure, which remarkably endow the electrocatalyst with high activity and stability.

**Abstract:**

Exploring highly active and stable transition metal-based bifunctional electrocatalysts has recently attracted extensive research interests for achieving high inherent activity, abundant exposed active sites, rapid mass transfer, and strong structure stability for overall water splitting. Herein, an interface engineering coupled with shell-protection strategy was applied to construct three-dimensional (3D) core‐shell Ni_x_S_y_@MnO_x_H_y_ heterostructure nanorods grown on nickel foam (Ni_x_S_y_@MnO_x_H_y_/NF) as a bifunctional electrocatalyst. Ni_x_S_y_@MnO_x_H_y_/NF was synthesized via a facile hydrothermal reaction followed by an electrodeposition process. The X-ray absorption fine structure spectra reveal that abundant Mn‐S bonds connect the heterostructure interfaces of Ni_x_S_y_@MnO_x_H_y_, leading to a strong electronic interaction, which improves the intrinsic activities of hydrogen evolution reaction and oxygen evolution reaction (OER). Besides, as an efficient protective shell, the MnO_x_H_y_ dramatically inhibits the electrochemical corrosion of the electrocatalyst at high current densities, which remarkably enhances the stability at high potentials. Furthermore, the 3D nanorod structure not only exposes enriched active sites, but also accelerates the electrolyte diffusion and bubble desorption. Therefore, Ni_x_S_y_@MnO_x_H_y_/NF exhibits exceptional bifunctional activity and stability for overall water splitting, with low overpotentials of 326 and 356 mV for OER at 100 and 500 mA cm^–2^, respectively, along with high stability of 150 h at 100 mA cm^–2^. Furthermore, for overall water splitting, it presents a low cell voltage of 1.529 V at 10 mA cm^–2^, accompanied by excellent stability at 100 mA cm^–2^ for 100 h. This work sheds a light on exploring highly active and stable bifunctional electrocatalysts by the interface engineering coupled with shell-protection strategy.
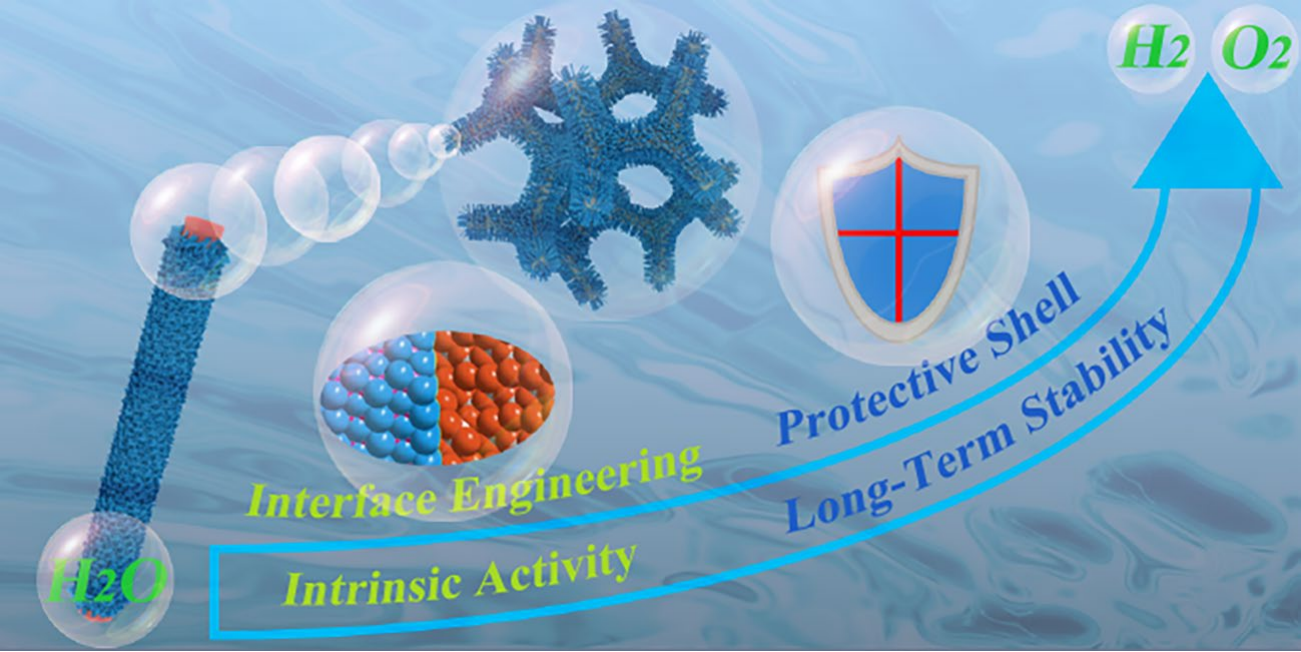

**Supplementary Information:**

The online version contains supplementary material available at 10.1007/s40820-022-00860-2.

## Introduction

The massive consumption of fossil fuels produces some serious negative effects, such as environmental pollution and energy crisis [1–3]. Therefore, developing renewable energy sources, such as hydrogen (H_2_) energy, has attracted great attention because H_2_ is considered a green energy alternative to fossil fuels due to its high energy density and environmentally friendly features [4, 5]. Among all kinds of H_2_ production, electrochemical water splitting, composed of cathodic hydrogen evolution reaction (HER) and anodic oxygen evolution reaction (OER), represents an ideal commercialized technology owing to the simple processing condition, zero carbon footprint, and high purity. However, low energy conversion efficiency for electrochemical water splitting greatly hinders its large‐scale application. Although Pt‐based and Ir/Ru‐based materials are regarded as the state‐of‐the‐art electrocatalysts for HER and OER, respectively, and their high cost and scarcity hamper their commercial applications [6–8]. As a consequence, it is an imperative call for researchers to design efficient electrocatalysts for water splitting by using non-noble materials.

Lately, transition metal-based electrocatalysts are being explored extensively due to their good activities and stabilities, such as transition‐metal oxides, chalcogenides, phosphides, and nitrides. In particular, resource‐rich nickel sulfides electrocatalysts, such as NiS [9], Ni_3_S_2_ [10, 11], NiS_2_ [12, 13], have been widely researched due to their high electronic conductivity and unique structural configuration. Unfortunately, these pure nickel sulfides electrocatalysts cannot satisfy the needs of commercial applications because of their insufficient activities and stabilities. Moreover, it is difficult for single‐component electrocatalysts to simultaneously own outstanding HER and OER activities as bifunctional electrocatalysts for overall water splitting due to the presence of different reaction intermediates in HER and OER processes [14]. As a result, substantial efforts have been devoted to exploring varieties of strategies to enhance the intrinsic activities of bifunctional electrocatalysts for overall water splitting, including foreign metal‐atom doping [15–17], interface engineering [18–20], and vacancy engineering [21, 22]. Among those strategies, it has been proposed that the interface engineering is a remarkably efficient route to boost both HER and OER intrinsic activities through coupling different active components for constructing heterostructures as bifunctional electrocatalysts. For example, Mu et al. reported an interface engineering of Co nanoparticles and Co_2_C nanowires with Co/Co_2_C heterostructures, requiring low overpotentials of 261 mV for OER and 96 mV for HER at 10 mA cm^–2^ in alkaline media, which can be attributed to that the Co and Co_2_C play a key role in HER and OER processes, respectively [23]. Besides, Ghosh et al. prepared bimetallic phosphide heterostructure of Ni_2_P–CuP_2_ on Ni foam‐graphene‐carbon nanotubes with ultralow overpotentials of 32 mV at 10 mA cm^–2^ for HER and 140 mV at 20 mA cm^–2^ for OER in alkaline mediums, exhibiting outstanding overall‐water‐splitting activities, because the synergistic effect in the Ni_2_P–CuP_2_ heterostructures accelerates the HER and OER kinetics [24]. In addition, three‐dimensional (3D) nanostructure is quite often used in the electrodes, for example, Li et al. presented a core‐shell electrocatalyst composed of 3D ordered macroporous Co(OH)_2_ cavity array‐encapsulated NiMo alloy on a flexible carbon cloth with a low cell voltage of 1.52 V at 10 mA cm^–2^ for overall water splitting, in which the 3D structure exposes abundant active sites and ensures a rapid mass transfer by accelerating the bubble evolution and desorption process [25].

Apart from the activity of electrocatalysts, their stabilities are another important indicator for commercial applications. The main reason for stability decreasing is that active components of electrocatalysts are changed in the HER or OER process, especially at high potentials. Many researchers have demonstrated that transition metal chalcogenides, nitrides, and phosphides can be easily oxidized to the corresponding metal oxides/(oxy)hydroxides in the OER process [26–29]. Accordingly, a shell-protection strategy can efficiently enhance the stabilities of the catalysts [30, 31]. For example, Bao et al. prepared a core‐shell structured electrocatalyst of ultrathin graphene shells encapsulating a uniform CoNi nanoalloy with high stability and activity for HER in acidic media, in which the carbon shells protect the CoNi nanoalloy from acid corrosion, leading to the improved stability [32]. In our previous work, hierarchical CoNi_2_S_4_@NiMn‐layered double hydroxide heterostructures were synthesized, where the NiMn‐layered double hydroxide acts as a protective layer that remarkably enhances the OER stability of the electrocatalyst at high potentials [33]. Besides, transition metal oxides and (oxy)hydroxides have attracted extensive research interest as the most common and stable OER catalysts [34–36]. For example, Sun et al. prepared nano‐architectured turbostratic δ-MnO_x_ on carbon nanotubes, which reaches 10 mA cm^–2^ at a low overpotential of 270 mV for OER [37]. Besides, Yang et al. reported a 2D NiFe LDH–Birnessite (MnO_2_·nH_2_O) hybrid, which shows outstanding catalytic activity (an overpotential of 258 mV at 10 mA cm^−2^) and excellent stability (20 h at 100 mA cm^–2^) for OER under a close to industrial hydrogen production condition (85 ℃ and 6 M KOH)) [38]. Accordingly, in order to synthesize excellent bifunctional HER and OER electrocatalysts for long‐term stability, transition metal oxides or (oxy)hydroxides as an OER active component can be applied as a shell to protect the core of HER active component. Furthermore, in situ growing electrocatalysts on nickel foam (NF) substrate remarkably improve the exposure and utilization of active sites owing to the characteristic of NF substrate, such as binder or adhesive-free, large surface area, and high electrical conductivity [39].

Herein, 3D core‐shell heterostructured Ni_x_S_y_@MnO_x_H_y_ nanorods grown on NF (Ni_x_S_y_@MnO_x_H_y_/NF) were successfully fabricated via a simple hydrothermal reaction and a subsequent electrodeposition process, in which the Ni_x_S_y_ is composed of Ni_3_S_2_ and NiS, while the MnO_x_H_y_ is a hybrid of MnOOH, Mn(OH)_2_, and MnO(OH)_2_. We demonstrate that proper modification of nickel sulfides-based electrocatalysts, i.e., MnO_x_H_y_ as a shell is combined with Ni_x_S_y_ core to construct heterostructures, can efficiently enhance both activities and stabilities of HER and OER. In addition, 3D nanorods on NF not only provide plentiful active sites, but also accelerate electrolyte access and bubble diffusion. As expected, the fabricated Ni_x_S_y_@MnO_x_H_y_/NF demonstrates outstanding bifunctional activity with low overpotentials of 270 mV for HER and 326 mV for OER at 100 mA cm^–2^ in 1.0 M KOH electrolyte, along with robust stability of 150 h for OER. Moreover, when it was applied as both anode and cathode for overall water splitting in the same alkaline media, the electrocatalyst affords 10 mA cm^–2^ at a low cell voltage of 1.529 V with excellent stability at 100 mA cm^–2^ for 100 h.

## Experimental Section

### Chemicals and Materials

Sulfur powder, hydrazine hydrate, cetyltrimethylammonium bromide (CTAB), MnSO_4_·H_2_O, CH_3_COONa, and concentrated HCl were purchased from the Sinopharm Chemical Reagent Co., Ltd. Commercial NF was provided by Lizhiyuan Battery Materials Co., Ltd. Pt/C (20 wt%) and RuO_2_ (99.95%) were obtained from Alfa Aesar.

### Preparation of Electrocatalysts

#### Preparation of 3D Ni_x_S_y_ Nanorods Grown on NF (Ni_x_S_y_/NF)

First, NF was ultrasonicated in 3.0 mol L^−1^ HCl solution, deionized (DI) water, and ethanol for 10 min, respectively, to remove the surface oxides and residues. Then, 2.2 mmol CTAB and 5 mL of hydrazine hydrate were dissolved in 60 mL of DI water. After transferring the above solution into a 100-mL Teflon‐lined stainless steel autoclave containing 4.4 mmol sulfur powder and a piece of NF (2.0 × 3.0 cm^2^), the autoclave was maintained at 160 ℃ for 12 h. Finally, Ni_x_S_y_/NF was obtained after being washed and dried.

#### Preparation of 3D Core‐shell Heterostructured Ni_x_S_y_@MnO_x_H_y_ Nanorods Grown on NF (Ni_x_S_y_@MnO_x_H_y_/NF)

Ni_x_S_y_@MnO_x_H_y_/NF was synthesized through a simple electrodeposition process in a three-electrode system, consisting of the Ni_x_S_y_/NF as the working electrode, a Pt wire as the counter electrode, and a saturated calomel electrode as the reference electrode. The anodic galvanostatic electrodeposition was conducted at 0.5 mA cm^−2^ for different time in 50 mL of electrolyte containing 7.5 mmol MnSO_4_·H_2_O and 15.0 mmol CH_3_COONa. After the electrodeposition process, Ni_x_S_y_@MnO_x_H_y_/NF was prepared after being rinsed and dried.

#### Preparation of Cotton-shaped MnO_x_H_y_ Grown on NF (MnO_x_H_y_/NF)

MnO_x_H_y_/NF was prepared with the same electrodeposition method, except that the Ni_x_S_y_/NF was replaced with bare NF.

### Physical Characterizations

The crystal structure was analyzed by X-ray diffraction (XRD) on a Rigaku D/Max 2400 X-ray diffractometer instrument. Scanning electron microscope (SEM, HITACHI UHR FE-SEM SU8200) and transmission electron microscopy (TEM, Talos F200S) were used to characterize the morphology. Inductively coupled plasma mass spectrometry (ICP-MS, Thermo, ICAP RQ) was carried out to detect the element content. X-ray photoelectron spectroscopy (XPS) measurements were carried out on a Thermo Scientific K-Alpha electron spectrometer with an exciting Al source (Kα = 1486.6 eV), in which all binding energies were corrected by referencing C 1 s peak (284.8 eV). X-ray absorption fine structure spectroscopy (XAFS) was conducted at the HXMA, SXRMB, and SGM beamlines at the Canadian Light Source (detail in supporting information).

### Electrochemical Measurements

All electrochemical measurements were performed at 25 ℃ in 1.0 M KOH electrolyte with a three-electrode system (Autolab PGSTAT302 N/FRA system), where NF with different electrocatalysts, a Hg/HgO electrode, and a carbon rod acted as the working, reference, and counter electrodes, respectively. All measured potentials were calibrated to the reversible hydrogen electrode (RHE) using *E*_RHE_ = *E*_Hg/HgO_ + 0.059 × pH + 0.098 (pH = 14). Linear sweep voltammetry (LSV) was conducted at a scan rate of 2 mV s^−1^, and electrochemical impedance spectroscopy (EIS) was obtained with a frequency range of 10^5^ to 0.1 Hz. For achieving the electrochemical active surface area (ECSA), cyclic voltammetry (CV) was tested at various scan rates. Chronopotentiometry curves were used to evaluate the stabilities of electrocatalysts. For overall-water-splitting tests, Ni_x_S_y_@MnO_x_H_y_/NF was applied as both anode and cathode in a two-electrode system.

## Result and Discussion

### Characterization of Morphology and Composition

**Fig. 1:**
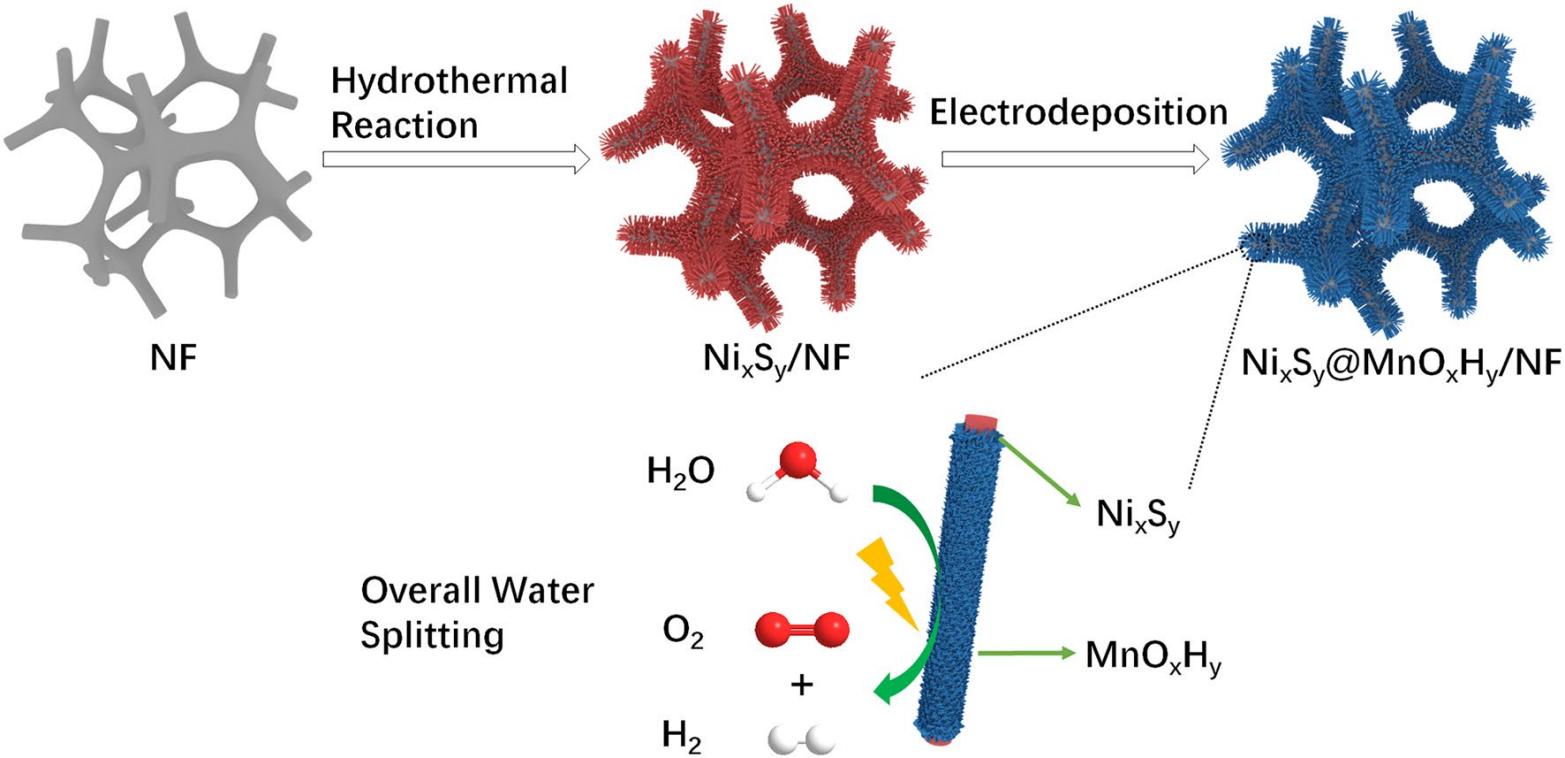
Schematic illustration of the synthesized process for Ni_x_S_y_@MnO_x_H_y_/NF

The fabricated procedure of Ni_x_S_y_@MnO_x_H_y_/NF is shown in Fig. 1. Initially, 3D Ni_x_S_y_ nanorods grown on NF were prepared via a facile hydrothermal sulfurization reaction, in which NF and sulfur powder, respectively, act as Ni and S sources in the presence of CTAB and hydrazine hydrate. Subsequently, through an electrodeposition process, Ni_x_S_y_/NF was uniformly covered with MnO_x_H_y_ to construct core–shell heterostructured Ni_x_S_y_@MnO_x_H_y_ nanorods. Besides, the mass loading of MnO_x_H_y_ can be optimized by adjusting the electrodeposition time.

**Fig. 2:**
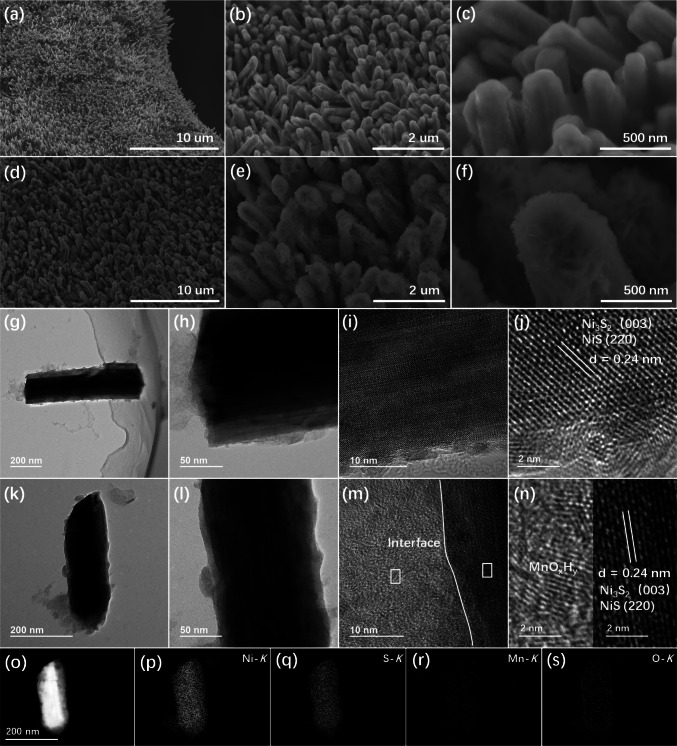
SEM images of **a–c** Ni_x_S_y_/NF and **d**–**f** Ni_x_S_y_@MnO_x_H_y_/NF. **g, h** TEM and **i, j** HRTEM images of Ni_x_S_y_ nanorod. **k, l** TEM and **m, n** HRTEM images of Ni_x_S_y_@MnO_x_H_y_ nanorod. **o–s** STEM image of Ni_x_S_y_@MnO_x_H_y_ nanorod and its corresponding EDX elemental mapping images

In order to investigate the morphology of as-prepared electrocatalysts, SEM characterization was utilized. As shown in Fig. 2a-c, 3D Ni_x_S_y_ nanorods were in situ grown successfully on the surface of NF via a facile hydrothermal reaction, compared with pure NF (Fig. S1a–c). Their high-magnified SEM images manifest that these nanorods possess a diameter of about 100–400 nm with relatively smooth surfaces. Such a 3D nanorod structure is beneficial to exposing abundant active sites and accelerating electrolyte contact and gas diffusion, leading to enhanced electrocatalytic activity. After the electrodeposition process, Fig. 2d–f shows that the entire surfaces of Ni_x_S_y_ nanorods are covered with many MnO_x_H_y_ nanosheets, which can be confirmed by the SEM image and the corresponding elemental mapping images (Fig. S2) of Ni_x_S_y_@MnO_x_H_y_/NF. As a comparison, MnO_x_H_y_ was electrodeposited on the surface of NF (Fig. S3a–c), showing its cotton-shaped structure when pure NF was applied as an electrodeposited substrate, which indicates that the composition of substrates has a great influence on the morphology of electrodeposited materials. Subsequently, TEM was applied to further analyze the morphological property. Figs. 2g–h and k–l present the nanorod structure of Ni_x_S_y_ and Ni_x_S_y_@MnO_x_H_y_, respectively. High-resolution TEM (HRTEM) images (Fig. 2i–j) of Ni_x_S_y_ nanorod demonstrate an interplanar spacing of 0.24 nm, corresponding to the (003) plane of Ni_3_S_2_ and (220) plane of NiS, which reveals that Ni_x_S_y_ consists of Ni_3_S_2_ and NiS. For Ni_x_S_y_@MnO_x_H_y_ nanorod (Fig. 2m), a clear interface is observed between Ni_x_S_y_ and MnO_x_H_y_. In addition, its HRTEM image (Fig. 2n) exhibits the crystalline MnO_x_H_y_ and the existence of lattice spacing of 0.24 nm which is from Ni_x_S_y_, confirming the existence of heterostructured Ni_x_S_y_@MnO_x_H_y_. Besides, Fig. S4 shows the scanning transmission electron microscopy (STEM) and corresponding energy dispersive X-ray (EDX) spectrum of Ni_x_S_y_@MnO_x_H_y_ nanorod, which also confirms the existence of Ni, S, Mn, and O in the Ni_x_S_y_@MnO_x_H_y_ nanorod, and the Mn/Ni molar ratio is 1:40.3. Meanwhile, the STEM and corresponding EDX element mapping images (Fig. 2o–s) of Ni_x_S_y_@MnO_x_H_y_ nanorod demonstrate the homogeneous spatial distribution of Ni, S, Mn, and O throughout the nanorod structure. Therefore, 3D core‐shell heterostructured Ni_x_S_y_@MnO_x_H_y_ nanorods were successfully grown on the surface of NF.

**Fig. 3:**
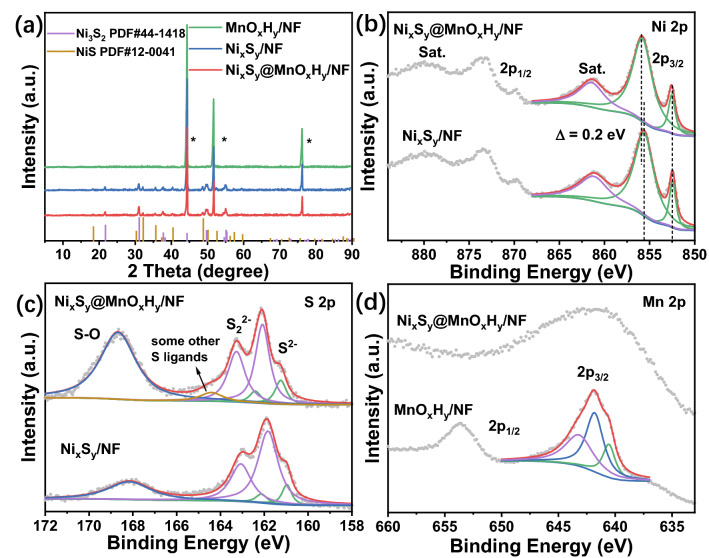
**a** XRD patterns of MnO_x_H_y_/NF, Ni_x_S_y_/NF, and Ni_x_S_y_@MnO_x_H_y_/NF (* indicates characteristic peaks of NF). High‐resolution XPS spectra of **b** Ni 2p and **c** S 2p for Ni_x_S_y_/NF and Ni_x_S_y_@MnO_x_H_y_/NF. High‐resolution XPS spectra of **d** Mn 2p for MnO_x_H_y_/NF and Ni_x_S_y_@MnO_x_H_y_/NF

The composition and crystalline structure of MnO_x_H_y_/NF, Ni_x_S_y_/NF, and Ni_x_S_y_@MnO_x_H_y_/NF were studied with XRD (Fig. 3a). Three obvious characteristic peaks marked by * can be ascribed to NF. The other peaks of Ni_x_S_y_/NF and Ni_x_S_y_@MnO_x_H_y_/NF match well with those of Ni_3_S_2_ (PDF#44–1418) and NiS (PDF#12–0041), revealing that Ni_x_S_y_ is a hybrid of Ni_3_S_2_ and NiS. However, after the electrodeposition procedure, no peaks of MnO_x_H_y_ can be observed in the XRD pattern of Ni_x_S_y_@MnO_x_H_y_/NF, which might be because the mass loading of the electrodeposited MnO_x_H_y_ is too low to generate diffusion peaks. Furthermore, XPS was applied to investigate the surface chemical composition and valence of as-prepared electrocatalysts. The XPS survey spectra (Fig. S5) manifest the existence of Ni, S, Mn, and O elements, consistent with the element mapping results. As shown in Fig. 3b, regarding Ni 2p XPS spectra of Ni_x_S_y_/NF and Ni_x_S_y_@MnO_x_H_y_/NF, the Ni 2p_3/2_ peak located at 852.5 eV is attributed to the Ni–Ni bonds in Ni_3_S_2_ [40, 41]. For Ni_x_S_y_/NF, the peak located at 855.6 eV belongs to Ni–S [42, 43]. When Ni_x_S_y_/NF was coupled with MnO_x_H_y_, the second peak of Ni 2p_3/2_ positively shifts from 855.6 to 855.8 eV, suggesting that some electrons can be transferred from Ni in the Ni_x_S_y_ by electrodepositing MnO_x_H_y_. The positive shift of 0.2 eV implies that MnO_x_H_y_ can result in the redistribution of charge density for Ni active sites in Ni_x_S_y_@MnO_x_H_y_/NF, further leading to enhanced electrocatalytic activities by optimizing the adsorption/desorption energy of intermedia. The peak at about 861.3 eV is related to the satellite, referred to as “Sat.” In Fig. 3c, the S 2p XPS spectra of Ni_x_S_y_/NF and Ni_x_S_y_@MnO_x_H_y_/NF were reasonably deconvoluted. The characteristic peak of S–O at approximately 168.4 eV comes from oxidized S species because of the air oxidation [44]. The first doublet of S 2p_3/2_ and S 2p_1/2_ at about 161.1 and 162.3 eV is associated with S^2−^, while the other doublet, corresponding to S_2_^2−^, appears at near 162.0/163.2 eV. In addition, for Ni_x_S_y_@MnO_x_H_y_/NF, the peak at 164.4 eV can be ascribed to some other S ligands, which are generated during the anodic electrodeposition process, where S-containing compositions, such as Ni_x_S_y_ and SO_4_^2−^, were transformed into some other S ligands. For Mn 2p_3/2_ (Fig. 3d), three peaks of MnO_x_H_y_/NF appear at 640.6, 641.8, and 643.19 eV, which are assigned to Mn^2+^, Mn^3+^, and Mn^4+^, respectively, signifying that MnO_x_H_y_ may be composed of Mn^2+^, Mn^3+^, and Mn^4+^ species [45]. Due to the partial overlapping of the Ni LMM Auger peaks and Mn 2p, the Mn 2p_3/2_ of Ni_x_S_y_@MnO_x_H_y_/NF is difficult to be deconvoluted exactly into multiple peaks. High‐resolution Mn 3s XPS spectra for MnO_x_H_y_/NF and Ni_x_S_y_@MnO_x_H_y_/NF were used to further analyze the Mn valence states. The peak separation (Δ*E*) of Mn 3s peaks can be used to distinguish Mn oxidation states [46]. Fig. S6 shows that the Δ*E* of MnO_x_H_y_/NF is 5.3 eV, indicating the existence of Mn_2_O_3_ in MnO_x_H_y_/NF. However, for Ni_x_S_y_@MnO_x_H_y_/NF, no obvious peaks appear, implying that manganese oxides are not contained in Ni_x_S_y_@MnO_x_H_y_/NF, owing to the same electrodeposition condition. Consequently, the MnO_x_H_y_ in Ni_x_S_y_@MnO_x_H_y_/NF may be a containing‐OH hybrid of Mn^2+^, Mn^3+^, and Mn^4+^. With regard to O 1s (Fig. S7), the peak at 529.6 eV is related to Mn–O, which is consistent with the result of Mn 3s spectra. The other two peaks are located at 531.0 and 531.8 eV, corresponding to –OH and –OOH, respectively. When the MnO_x_H_y_ was electrodeposited on Ni_x_S_y_/NF substrate, the electrodeposited potentials are much lower than the electrochemical oxidation potential (1.37 V) of Ni_x_S_y_/NF (Figs. S8 and S9), which results in no formation of nickel (oxy)hydroxide in the electrodeposition process. Therefore, –OH and –OOH are attributed to Mn species. According to the above results of Mn 2p, Mn 3s and O 1s, the MnO_x_H_y_ in MnO_x_H_y_/NF may consist of Mn(OH)_2_, MnOOH, Mn_2_O_3_, and MnO(OH)_2_, while the MnO_x_H_y_ in Ni_x_S_y_@MnO_x_H_y_/NF may be composed of Mn(OH)_2_, MnOOH, and MnO(OH)_2_. In brief, MnO_x_H_y_ was successfully coupled to Ni_x_S_y_/NF to form Ni_x_S_y_@MnO_x_H_y_/NF via an electrodeposition method, while an electronic coupling interaction exists in the heterostructure interfaces.

**Fig. 4:**
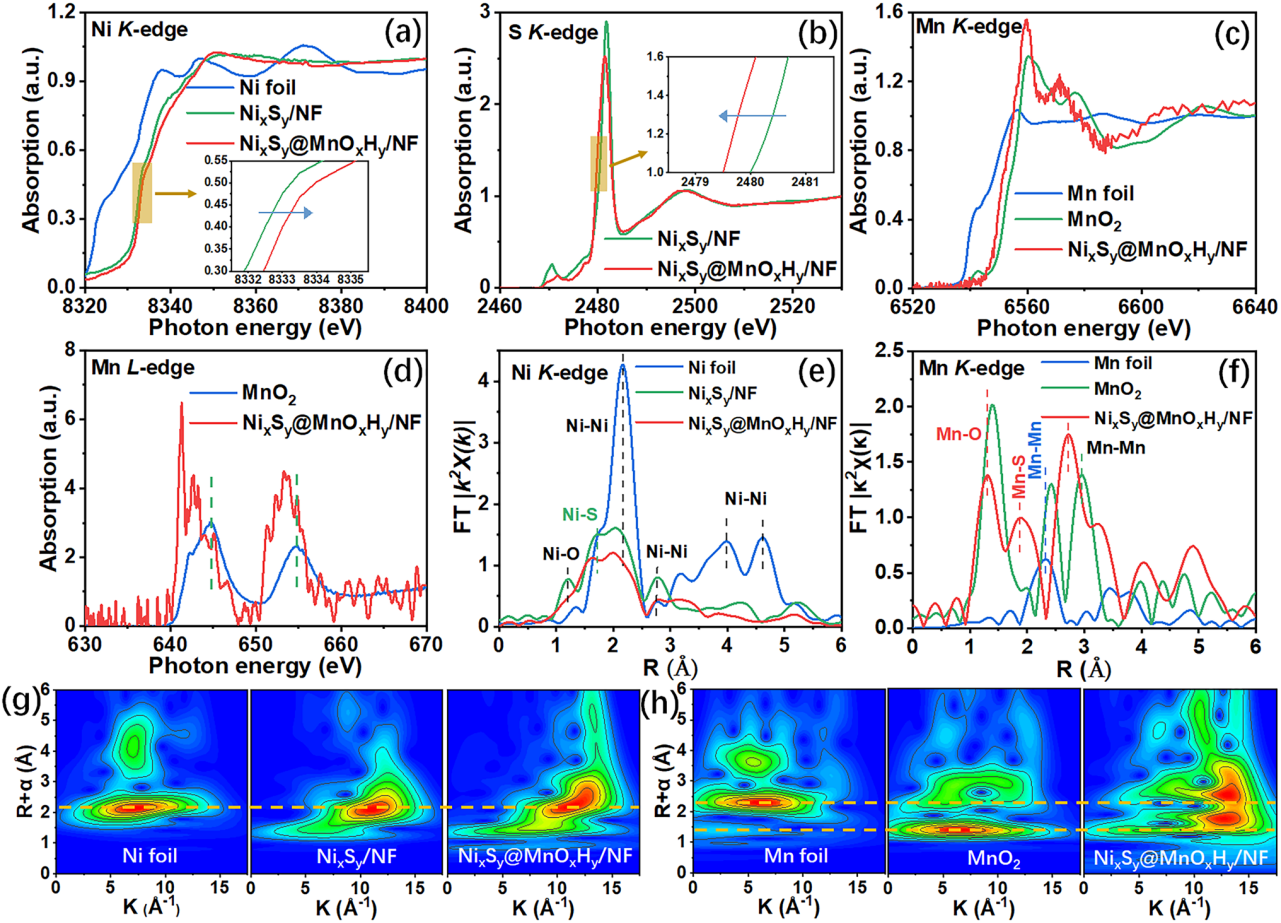
**a** Normalized Ni *K*-edge XANES spectra of Ni_x_S_y_/NF, Ni_x_S_y_@MnO_x_H_y_/NF, and Ni foil. **b** Normalized S *K*-edge XANES spectra of Ni_x_S_y_/NF and Ni_x_S_y_@MnO_x_H_y_/NF. **c** Normalized Mn *K*-edge XANES spectra of Ni_x_S_y_@MnO_x_H_y_/NF, Ni foil, and MnO_2_ standard sample. **d** Normalized Mn *L*-edge XANES spectra of MnO_2_ standard sample and Ni_x_S_y_@MnO_x_H_y_/NF. **e** Ni *K*-edge FT‐EXAFS oscillation functions k^2^χ(k) of Ni_x_S_y_/NF, Ni_x_S_y_@MnO_x_H_y_/NF, and Ni foil. **f** Mn *K*-edge FT‐EXAFS oscillation functions k^2^χ(k) of Ni_x_S_y_@MnO_x_H_y_/NF, Ni foil, and MnO_2_ standard sample. **g** Corresponding wavelet transform of Ni *K*-edge EXAFS oscillation for Ni foil, Ni_x_S_y_/NF, and Ni_x_S_y_@MnO_x_H_y_/NF. **h** Corresponding wavelet transform of Mn *K*-edge EXAFS oscillation for Mn foil, MnO_2_ standard sample, and Ni_x_S_y_@MnO_x_H_y_/NF

Moreover, XAFS was conducted to further investigate the electronic states and coordination environments of as-prepared electrocatalysts [47]. The X-ray absorption near edge structure (XANES) spectra of Ni, S, and Mn *K*-edges of Ni_x_S_y_/NF and Ni_x_S_y_@MnO_x_H_y_/NF are shown in Fig. 4a–c, respectively. The Ni *K*‐edge XANES spectrum of Ni_x_S_y_/NF is very close to that of Ni_x_S_y_@MnO_x_H_y_/NF. The much more positive energy position of the white line of Ni_x_S_y_/NF and Ni_x_S_y_@MnO_x_H_y_/NF than that of Ni foil manifests that Ni_x_S_y_/NF and Ni_x_S_y_@MnO_x_H_y_/NF have oxidized Ni. Fig. 4b shows that for the pre-edge of S *K*‐edge, the peak position of Ni_x_S_y_@MnO_x_H_y_/NF shifts to the higher photon energy than Ni_x_S_y_/NF, which is ascribed to that an electronic interaction exists between Ni_x_S_y_ and MnO_x_H_y_. Additionally, the magnified Ni and S *K*‐edges spectrum (insets in Fig. 4a–b) show that Ni_x_S_y_@MnO_x_H_y_/NF possesses higher Ni *K*‐edge energy and lower S *K*‐edge energy than Ni_x_S_y_/NF, which can be attributed to that the electrodeposited MnO_x_H_y_ leads to the electron transfer from Ni to S for Ni_x_S_y_@MnO_x_H_y_/NF. Hence, an electronic interaction exists between Ni_x_S_y_ and MnO_x_H_y_ for Ni_x_S_y_@MnO_x_H_y_/NF, which is coincident with the XPS results. For Mn *K*-edge (Fig. 4c), the shape of the post-edge of Ni_x_S_y_@MnO_x_H_y_/NF is different to that of the MnO_2_ standard sample, implying that the MnO_x_H_y_ in Ni_x_S_y_@MnO_x_H_y_/NF and MnO_2_ own different crystal structures. Besides, the Mn *K*‐edge position of Ni_x_S_y_@MnO_x_H_y_/NF is much higher than that of Mn foil, but lower than that of MnO_2_ standard sample, verifying that the average valence of Mn in Ni_x_S_y_@MnO_x_H_y_/NF is lower than + 4. More evidence is shown in Mn *L*-edge spectra (Fig. 4d). The peak positions of MnO_2_ are located in much higher photon energies than those of Ni_x_S_y_@MnO_x_H_y_/NF, indicating the Mn valence in Ni_x_S_y_@MnO_x_H_y_/NF is lower than + 3, in agreement with the result of Mn *K*‐edge spectra. In addition, Fourier transformed extended X-ray absorption fine structure (FT‐EXAFS) spectra were used to further characterize the coordinative geometry. Fig. 4e shows Ni *K*-edge FT‐EXAFS oscillation functions k^2^χ(k) of Ni_x_S_y_/NF, Ni_x_S_y_@MnO_x_H_y_/NF, and Ni foil, in which Ni_x_S_y_/NF and Ni_x_S_y_@MnO_x_H_y_/NF present similar Ni coordinative geometry with two main peaks of Ni–S at about 1.7 Å and Ni–Ni at around 2.0 Å [48]. However, the peak intensity of Ni–S of Ni_x_S_y_@MnO_x_H_y_/NF is lower than that of Ni_x_S_y_/NF, revealing the decrease in the Ni–S coordination number in Ni_x_S_y_@MnO_x_H_y_/NF, which could be attributed to the formation of Mn–S bonds. Besides, the appearance of Ni–O peak at 1.2 Å may be due to the oxidation of Ni_x_S_y_ in air. For Mn *K*-edge FT‐EXAFS oscillation functions k^2^χ(k) (Fig. 4f), an obvious Mn–S peak appears at 1.9 Å for Ni_x_S_y_@MnO_x_H_y_/NF, further confirming the existence of Mn–S bonds. In addition, two peaks located at 1.3 and 2.7 Å can be assigned to the Mn–O and Mn–Mn bonds, respectively. In the meantime, two main peaks of the MnO_2_ standard sample, related to the Mn–Mn bonds, are located at 2.4 and 3.0 Å, signifying MnO_x_H_y_ and MnO_2_ have different crystal structures, corresponding to the Mn *K*-edge near edge spectra result (Fig. 4c). The corresponding wavelet transform of Ni and Mn *K*-edge EXAFS oscillations was carried out to further present the atomic dispersion [49]. For Ni *K*-edge EXAFS (Fig. 4g), the maximum intensity positions of Ni foil and Ni_x_S_y_/NF are located at about 2.2 Å, which can be ascribed to the contribution of Ni–Ni bonds. However, the maximum intensity position of Ni_x_S_y_@MnO_x_H_y_/NF, associated with Ni–Ni bonds, positively shift to about 2.3 Å, which may be attributed to the Ni–Mn contribution from the Ni_x_S_y_@MnO_x_H_y_ heterostructure interfaces. Fig. 4h shows that Ni_x_S_y_@MnO_x_H_y_/NF, Mn foil, and MnO_2_ standard sample display different Mn atom dispersion, in accord with the Mn *K*-edge FT‐EXAFS result. For Mn foil, two maximum intensities at 2.3 and 3.6 Å come from the Mn–Mn bonds, while for the MnO_2_ standard sample, two maximum intensities at 1.4 and 2.7 Å are ascribed to the Mn–O and Mn–Mn bonds, respectively. Besides, two maximum intensities of Ni_x_S_y_@MnO_x_H_y_/NF, located at 1.8 and 2.6 Å, are assigned to the Mn–O and Mn–Mn bonds, respectively. For Ni_x_S_y_@MnO_x_H_y_/NF, the positive shift of the maximum intensity position, associated with the Mn–O bond, is dominated by the Mn–S contribution from the interfaces. Therefore, the two crystal phases (Ni_x_S_y_ and MnO_x_H_y_) are connected through Mn–S bonds to construct heterostructures with electronic coupling effects in Ni_x_S_y_@MnO_x_H_y_/NF, which could modulate surface electronic structure to optimize adsorption/desorption energies of reaction intermediates during the HER/OER process, leading to enhanced electrocatalytic activity for water splitting.

### Electrocatalytic OER Measurement

**Fig. 5:**
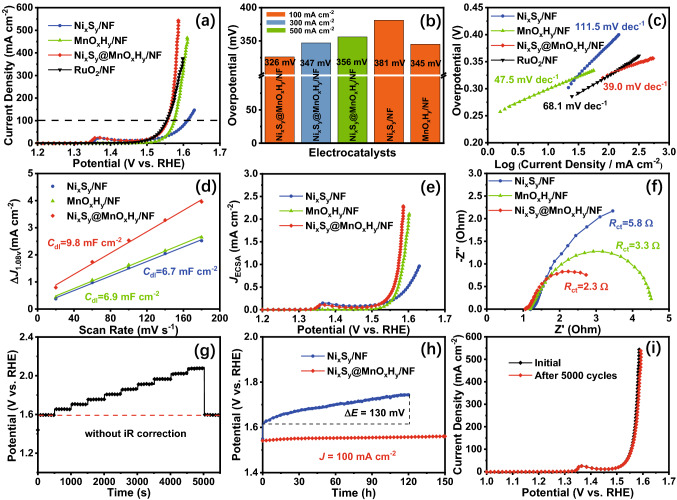
**a** LSV curves of Ni_x_S_y_/NF, MnO_x_H_y_/NF, Ni_x_S_y_@MnO_x_H_y_/NF, and RuO_2_/NF for OER in 1.0 M KOH electrolyte. **b** Comparison of OER activities for as-prepared electrocatalysts at different high current densities. **c** Corresponding Tafel plots of the electrocatalysts and RuO_2_/NF. **d** The estimated *C*_dl_, **e** corresponding LSV curves normalized by ECSA, and **f** Nyquist plots at a potential of 1.53 V for the electrocatalysts. **g** Multi‐current process of Ni_x_S_y_@MnO_x_H_y_/NF at 50 mA cm^–2^ per stair from 50 to 500 mA cm^–2^. **h** Chronopotentiometry curves of Ni_x_S_y_/NF and Ni_x_S_y_@MnO_x_H_y_/NF at 100 mA cm^–2^. **i** Comparison of LSV curves for Ni_x_S_y_@MnO_x_H_y_/NF before and after the 5000‐cycle stability

The OER performance of as-synthesized electrocatalysts was studied with a standard there-electrode configuration in 1.0 M KOH electrolyte, as shown in Fig. 5. To begin with, the OER activities of Ni_x_S_y_@MnO_x_H_y_/NF with different electrodeposition time were investigated. Fig. S9 shows that the catalyst prepared with 150 s electrodeposition time can achieve the highest OER activity. In addition, for comparison, LSV curves of Ni_x_S_y_/NF, MnO_x_H_y_/NF, and RuO_2_/NF were collected in the same condition (Fig. 5a). The OER activity of Ni_x_S_y_@MnO_x_H_y_/NF is similar to that of noble metal RuO_2_/NF under the low‐current‐density range, but higher than that of RuO_2_/NF under the high‐current‐density range, testifying that Ni_x_S_y_@MnO_x_H_y_/NF owns an excellent OER activity. Besides, it also presents better OER activity than Ni_x_S_y_/NF and MnO_x_H_y_/NF, indicating that a synergistic effect between Ni_x_S_y_ and MnO_x_H_y_ endows Ni_x_S_y_@MnO_x_H_y_/NF with the enhanced OER activity. Fig. 5b compares OER activities of as‐prepared electrocatalysts at different high current densities. Ni_x_S_y_@MnO_x_H_y_/NF can afford 100, 300, and 500 mA cm^–2^ at low overpotentials of 326, 347, and 356 mV, respectively, whereas Ni_x_S_y_/NF and MnO_x_H_y_/NF need overpotentials of 381 and 345 mV to reach 100 mA cm^–2^, respectively. Furthermore, Tafel slopes of electrocatalysts are used to study the reaction kinetics and intrinsic activity. Fig. 5c shows that the Tafel slope of Ni_x_S_y_@MnO_x_H_y_/NF is 39.0 mV dec^–1^, smaller than those of Ni_x_S_y_/NF (111.5 mV dec^–1^), MnO_x_H_y_/NF (47.5 mV dec^–1^), and RuO_2_/NF (68.1 mV dec^–1^), suggesting that Ni_x_S_y_@MnO_x_H_y_/NF secures the fast reaction kinetics and outstanding inherent activity for OER, which enables Ni_x_S_y_@MnO_x_H_y_/NF to outperform most reported non‐noble metal OER electrocatalysts (Table S1).

Moreover, the electrochemical active surface area (ECSA), which has a linear relation with the double-layer capacitance (*C*_dl_), is another important parameter to reflect the activities of electrocatalysts (Fig. S10). As shown in Fig. 5d, Ni_x_S_y_@MnO_x_H_y_/NF attains a larger *C*_dl_ (9.8 mF cm^−2^) than Ni_x_S_y_/NF (6.7 mF cm^−2^), MnO_x_H_y_/NF (6.9 mF cm^−2^), revealing that Ni_x_S_y_@MnO_x_H_y_/NF has the largest ECSA among as-synthesized electrocatalysts. In other words, more exposed active sites are available on the surface of Ni_x_S_y_@MnO_x_H_y_/NF, manifesting that the fabricated heterostructures are beneficial to creating more electrochemically accessible active sites [50, 51]. Moreover, the ECSA was utilized to calculate the intrinsic activity, and the corresponding LSV curves based on the specific current density (*J*_ECSA_) are shown in Fig. 5e. Apparently, Ni_x_S_y_@MnO_x_H_y_/NF achieves the higher inherent OER activity than Ni_x_S_y_/NF and MnO_x_H_y_/NF, which can be ascribed to the synergy in the heterostructure interfaces. In addition, the EIS was conducted to further assess the electrode kinetics and electron transport capability of these samples. Their Nyquist plots (Fig. 5f) demonstrate that the charge‐transfer resistance (*R*_ct_) of Ni_x_S_y_@MnO_x_H_y_/NF is 2.3 Ω, smaller than those of Ni_x_S_y_/NF (5.8 Ω) and MnO_x_H_y_/NF (3.3Ω), signifying that Ni_x_S_y_@MnO_x_H_y_/NF owns an enhanced reaction kinetics toward OER, leading to the rapid electron transfer, guaranteed by the synergistic effect in heterostructures, which is consistent with the result of Tafel plots.

Stability is an important metric for examining commercial applications. Firstly, the multi‐current process of Ni_x_S_y_@MnO_x_H_y_/NF was studied to evaluate the robust stability (Fig. 5g). The current density started at 50 mA cm^–2^, while the potential responded fast and remained stable for 500 s. Then, the current density increased to 500 mA cm^–2^ with 50 mA cm^–2^ per stair. At last, when it went back to the initial 50 mA cm^–2^, it was still stable, manifesting the robust stability of Ni_x_S_y_@MnO_x_H_y_/NF, whereafter the chronopotentiometry was applied to compare the stabilities of Ni_x_S_y_/NF and Ni_x_S_y_@MnO_x_H_y_/NF (Fig. 5h). Ni_x_S_y_@MnO_x_H_y_/NF can almost maintain its potential at 100 mA cm^–2^ for 150 h, whereas the potential of Ni_x_S_y_/NF decreases by 130 mV within 120 h, which verifies that Ni_x_S_y_@MnO_x_H_y_/NF owns an excellent OER stability owing to the MnO_x_H_y_ shell. In the literature, to evaluate the stability, most reported non‐noble metal OER electrocatalysts are usually characterized at low current densities (< 100 mA cm^−2^) or short test time (< 100 h), which is far away from commercial requirements. As can be seen, the stability of Ni_x_S_y_@MnO_x_H_y_/NF outperforms almost all of non‐noble metal OER electrocatalysts (Table S1). At the same time, the instability of Ni_x_S_y_ and the high stability of Ni_x_S_y_@MnO_x_H_y_/NF under high anodic oxidized potentials prove that the MnO_x_H_y_ as a protective shell can efficiently inhibit the electrochemical corrosion under high anodic oxidized potentials, leading to the remarkably improved OER stability. Hence, those results demonstrate the great potential of as‐prepared Ni_x_S_y_@MnO_x_H_y_/NF for large‐scale applications. Additionally, after 5000 cycles, the collected LSV curve of Ni_x_S_y_@MnO_x_H_y_/NF almost coincides with its initial curve (Fig. 5i), further confirming its consistent high OER durability.

### Electrocatalytic HER Measurement

**Fig. 6:**
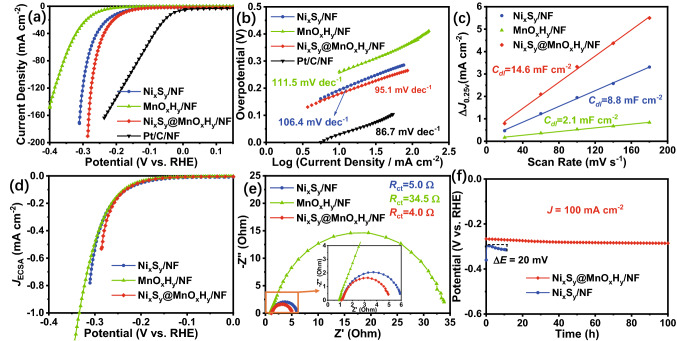
**a** LSV curves and **b** the corresponding Tafel plots of Ni_x_S_y_/NF, MnO_x_H_y_/NF, Ni_x_S_y_@MnO_x_H_y_/NF, and Pt/C/NF for HER in 1.0 M KOH electrolyte. **c** The estimated *C*_dl_, **d** corresponding LSV curves normalized by ECSA, and **e** Nyquist plots at a potential of -0.2 V for the electrocatalysts. **f** Chronopotentiometry curves of Ni_x_S_y_@MnO_x_H_y_/NF and Ni_x_S_y_/NF at 100 mA cm^–2^

Apart from the OER performance, the HER performance of the electrocatalysts was also assessed in 1.0 M KOH electrolyte. Fig. 6a shows LSV curves of as-fabricated electrocatalysts and Pt/C/NF. As expected, the HER activity of Ni_x_S_y_@MnO_x_H_y_/NF is better than those of Ni_x_S_y_/NF and MnO_x_H_y_/NF, as well as close to that of Pt/C/NF, which is attributed to the synergistic effect enabled by the electronic interaction between Ni_x_S_y_ and MnO_x_H_y_. Besides, Ni_x_S_y_@MnO_x_H_y_/NF requires overpotentials of 179 and 270 mV to achieve 10 and 100 mA cm^–2^, respectively, exhibiting efficient HER activity, which can compare favorably with most non-noble metal electrocatalysts for HER (Table S2). As shown in Fig. 6b, Ni_x_S_y_@MnO_x_H_y_/NF owns a smaller Tafel slope (95.1 mV dec^–1^) than those of Ni_x_S_y_/NF (106.4 mV dec^–1^) and MnO_x_H_y_/NF (111.5 mV dec^–1^), implying that Ni_x_S_y_@MnO_x_H_y_/NF possesses favorable reaction kinetics for HER.

The ECSA of Ni_x_S_y_@MnO_x_H_y_/NF is measured according to the *C*_dl_ (Figs. S11 and 6c). The *C*_dl_ of Ni_x_S_y_@MnO_x_H_y_/NF is 14.6 mF cm^−2^, larger than those of Ni_x_S_y_/NF (8.8 mF cm^−2^) and MnO_x_H_y_/NF (2.1 mF cm^−2^), which indicates that Ni_x_S_y_@MnO_x_H_y_/NF owns the largest ECSA with the most exposed active sites among as-prepared electrocatalysts. Subsequently, the *J*_ECSA_ is also evaluated to study the intrinsic activity. Fig. 6d demonstrates that Ni_x_S_y_@MnO_x_H_y_/NF possesses higher inherent activity than Ni_x_S_y_/NF and MnO_x_H_y_/NF, which is ascribed to the electronic coupling effect in the heterostructured Ni_x_S_y_@MnO_x_H_y_/NF. Furthermore, EIS was also used to study the electrode kinetics toward HER. As shown in Fig. 6e, Ni_x_S_y_@MnO_x_H_y_/NF exhibits smaller *R*_ct_ (4.0 Ω) than Ni_x_S_y_/NF (5.0 Ω) and MnO_x_H_y_/NF (34.5 Ω), suggesting the rapid reaction kinetics of Ni_x_S_y_@MnO_x_H_y_/NF, in agreement with the result of Tafel plots. In addition, Fig. 6f shows the chronopotentiometry curves of Ni_x_S_y_@MnO_x_H_y_/NF and Ni_x_S_y_/NF at 100 mA cm^–2^. After 10 h, the potential of Ni_x_S_y_/NF increases by 20 mV, whereas the potential of Ni_x_S_y_@MnO_x_H_y_/NF can remain mostly unchanged for 100 h, testifying that due to the MnO_x_H_y_ shell, Ni_x_S_y_@MnO_x_H_y_/NF owns excellent HER stability at high current densities, which surpasses most recently reported non‐noble metal HER electrocatalysts (Table S2).

### Electrocatalytic Overall‐Water‐Splitting Measurement

**Fig. 7:**
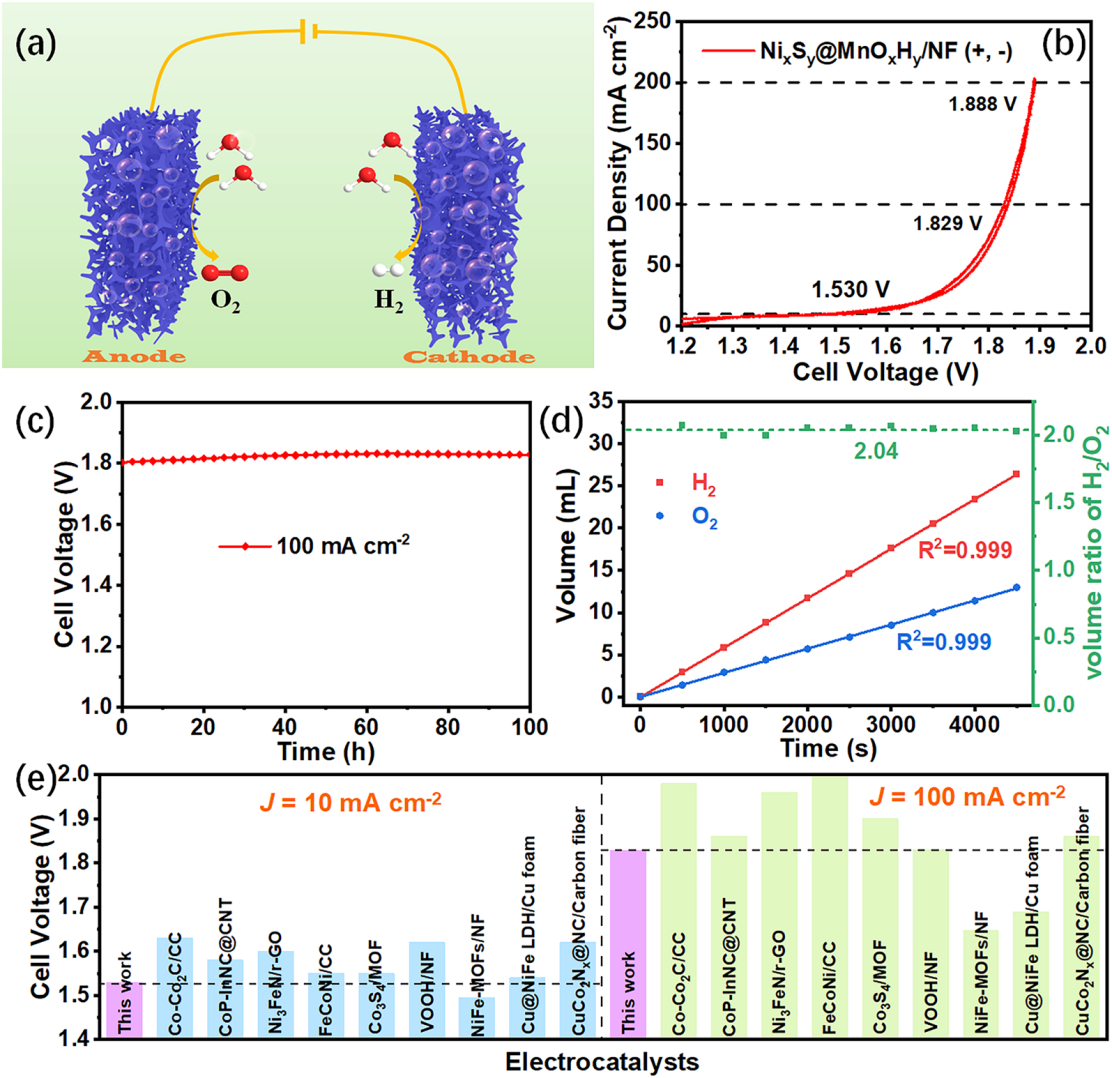
**a** Schematic illustration of the two-electrode system for overall water splitting. **b** CV curve and **c** Chronopotentiometry curve of Ni_x_S_y_@MnO_x_H_y_/NF as both the anode and cathode for overall water splitting. **d** The time-dependent volume of H_2_ and O_2_ collected by the drainage method, and the corresponding volume ratio of H_2_/O_2_. **e** Comparison of cell voltages for recently different electrocatalysts (Table S3) at 10 and 100 mA cm^–2.^

Inspired by the outstanding OER and HER performance, Ni_x_S_y_@MnO_x_H_y_/NF as a bifunctional electrocatalyst was applied as both the anode and cathode for overall water splitting in 1.0 M KOH electrolyte (Fig. 7a). As shown in Fig. 7b, Ni_x_S_y_@MnO_x_H_y_/NF attains superior activity for overall water splitting with a low cell voltage of 1.530 V at 10 mA cm^–2^. Besides, it only needs cell voltages of 1.829 and 1.888 V to drive 100 and 200 mA cm^–2^, respectively. Fig. 7c shows its cell voltage can remain almost unchanged at the high current density of 100 mA cm^–2^ for 100 h. Besides, its chronoamperometry curve also indicates its excellent stability at the cell voltage of 1.83 V for 200 h (Fig. S12). Currently, many researchers reported their stability tests at low current densities, such as 10, 20, and 50 mA cm^–2^ [23, 25, 52]. However, such low current densities of water splitting are not significant enough to meet the practical applications. As a consequence, electrocatalytic activities at the high current density region (≥ 100 mA cm^–2^) are considered for possible industrial applications. The excellent stability at 100 mA cm^–2^ for Ni_x_S_y_@MnO_x_H_y_/NF testifies its potential industrial applications, outperforming almost all of the recently reported non‐noble metal electrocatalysts for overall water splitting (Table S3). Furthermore, the faradaic efficiency (FE) of Ni_x_S_y_@MnO_x_H_y_/NF was measured to assess the efficiency of H_2_ and O_2_ production via a simple drainage method (Fig. S13). Fig. 7d demonstrates the time-dependent volumes of H_2_ and O_2_ collected by the drainage method, and the corresponding volume ratio (2.04:1) of H_2_/O_2_ is very close to the theoretical volume ratio of 2:1, which indicates that Ni_x_S_y_@MnO_x_H_y_/NF has a high FE of almost 100% for overall water splitting. In addition, the overall-water-splitting activity of Ni_x_S_y_@MnO_x_H_y_/NF is compared with recently reported different non-noble metal bifunctional electrocatalysts at 10 and 100 mA cm^–2^ (Fig. 7e and Table S3), signifying that the bifunctional activity of Ni_x_S_y_@MnO_x_H_y_/NF outperforms those of most recently non-noble metal bifunctional electrocatalysts. The superior activity and stability with low price enable Ni_x_S_y_@MnO_x_H_y_/NF to become a potential candidate for large-scale applications.

### High‐Performance Origination Analysis

To further investigate the origination of the high bifunctional activity and stability of Ni_x_S_y_@MnO_x_H_y_/NF, its anodic and cathodic composition and chemical valences after the stability test at 100 mA cm^–2^ for 100 h were analyzed by XRD and XPS. As shown in Fig. S14a, XRD patterns of initial Ni_x_S_y_@MnO_x_H_y_/NF and the corresponding anode and cathode exhibit that after the stability test, the peaks, related to Ni_3_S_2_, remain unaltered, while the peaks of NiS disappear, which implies that NiS may be unstable in both HER and OER processes or dissolved in the alkaline solution. Then, the Ni content of the electrolyte after the stability measurement was detected by ICP‐MS, and the corresponding result indicates almost no Ni content exits in the electrolyte. In the meantime, after Ni_x_S_y_@MnO_x_H_y_/NF was immersed in 1.0 M KOH electrolyte for 100 h, its XRD pattern (Fig. S14b) shows the existence of both NiS and Ni_3_S_2_. The above results testify that NiS may be transformed into amorphous Ni species (NiO_x_H_y_). Furthermore, high‐resolution XPS spectra (Fig. S15) of Ni 2p, Mn 2p, S 2p, and O 1s for the cathode and anode demonstrate that chemical valences of these elements can be stable even after the stability test, manifesting that Ni_3_S_2_ and MnO_x_H_y_ maintain exceptionally long-term stability at high current densities. Therefore, it can be concluded that the MnO_x_H_y_ as an efficient protective shell can dramatically enhance the stability at high current densities in HER and OER processes. Meanwhile, Ni_3_S_2_/NiO_x_H_y_@MnO_x_H_y_ are applied as the electrocatalytic active phases to effectively facilitate the HER and OER processes.

## Conclusions

In summary, 3D core‐shell Ni_x_S_y_@MnO_x_H_y_ heterostructure nanorods were successfully grown on the surface of NF by a simple two‐step method of hydrothermal and electrodeposition process. The MnO_x_H_y_ and Ni_x_S_y_ are integrated to form heterostructures with enriched Mn–S bonds, leading to a strong electronic interaction. The synergistic effect between MnO_x_H_y_ and Ni_x_S_y_ efficiently accelerates the kinetics and enhances the charge transfer in HER and OER processes. Besides, the MnO_x_H_y_ is applied as an efficient protective shell to remarkably improve the stability for water splitting. Moreover, the 3D nanorod structure is beneficial to exposing abundant active sites and accelerating the electrolyte access and bubbles diffusion. Therefore, as‐constructed Ni_x_S_y_@MnO_x_H_y_/NF exhibits outstanding bifunctional activity and stability for overall water splitting in alkaline solution, compared to recently reported non-noble metal electrocatalysts. For OER, Ni_x_S_y_@MnO_x_H_y_/NF only needs low overpotentials of 326 and 356 mV to afford 100 and 500 mA cm^–2^, respectively, with outstanding stability at 100 mA cm^–2^ for 150 h, while for HER, it can achieve 10 and 100 mA cm^–2^ at overpotentials of 179 and 270 mV, respectively. Moreover, it requires a low cell voltage of 1.529 V at 10 mA cm^–2^ for overall water splitting with excellent stability at 100 mA cm^–2^ for 100 h. Accordingly, such superior performance with low prices enables Ni_x_S_y_@MnO_x_H_y_/NF to become a promising candidate for large-scale applications. Furthermore, the interface engineering coupled with the shell-protection strategy sheds a light on developing highly efficient bifunctional electrocatalysts.

## Supplementary Information

Below is the link to the electronic supplementary material.

Supplementary file1 (PDF 3005 kb)

## References

[1] Zhao CX, Liu JN, Wang J, Ren D, Li BQ (2021). Recent advances of noble-metal-free bifunctional oxygen reduction and evolution electrocatalysts. Chem. Soc. Rev..

[2] Yao L, Lin J, Chen Y, Li X, Wang D (2021). Supramolecular-mediated ball-in-ball porous carbon nanospheres for ultrafast energy storage. InfoMat.

[3] Jiang WJ, Tang T, Zhang Y, Hu JS (2020). Synergistic modulation of non-precious-metal electrocatalysts for advanced water splitting. Acc. Chem. Res..

[4] Zhou Z, Pei Z, Wei L, Zhao SL, Jian X (2020). Electrocatalytic hydrogen evolution under neutral pH conditions: current understandings, recent advances, and future prospects. Energy Environ. Sci..

[5] Chen J, Chen H, Yu T, Li R, Wang Y (2021). Recent advances in the understanding of the surface reconstruction of oxygen evolution electrocatalysts and materials development. Electrochem. Energy Rev..

[6] Zhao T, Wang Y, Karuturi S, Catchpole K, Zhang Q (2020). Design and operando/in situ characterization of precious-metal-free electrocatalysts for alkaline water splitting. Carbon Energy.

[7] Zhang W, Chao Y, Zhang W, Zhou J, Lv F (2021). Emerging dual-atomic-site catalysts for efficient energy catalysis. Adv. Mater..

[8] Wu H, Feng C, Zhang L, Zhang J, Wilkinson DP (2021). Non-noble metal electrocatalysts for the hydrogen evolution reaction in water electrolysis. Electrochem. Energy Rev..

[9] Chen JS, Ren J, Shalom M, Fellinger T, Antonietti M (2016). Stainless steel mesh-supported NiS nanosheet array as highly efficient catalyst for oxygen evolution reaction. ACS Appl. Mater. Interfaces.

[10] Tong M, Wang L, Yu P, Tian C, Liu X (2018). Ni_3_S_2_ nanosheets in situ epitaxially grown on nanorods as high active and stable homojunction electrocatalyst for hydrogen evolution reaction. ACS Sustain. Chem. Eng..

[11] Zhou W, Wu XJ, Cao X, Huang X, Tan C (2013). Ni_3_S_2_ nanorods/Ni foam composite electrode with low overpotential for electrocatalytic oxygen evolution. Energy Environ. Sci..

[12] Karakaya C, Solati N, Savacı U, Keleş E, Turan S (2020). Mesoporous thin-film NiS_2_ as an idealized pre-electrocatalyst for a hydrogen evolution reaction. ACS Catal..

[13] Wang P, Wang T, Qin R, Pu Z, Zhang C (2022). Swapping catalytic active sites from cationic Ni to anionic S in nickel sulfide enables more efficient alkaline hydrogen generation. Adv. Energy Mater..

[14] Wang Y, Qiu W, Song E, Gu F, Zheng Z (2017). Adsorption-energy-based activity descriptors for electrocatalysts in energy storage applications. Natl. Sci. Rev..

[15] Xiong Q, Wang Y, Liu PF, Zheng LR, Wang G (2018). Cobalt covalent doping in MoS_2_ to induce bifunctionality of overall water splitting. Adv. Mater..

[16] Su H, Song S, Li S, Gao Y, Ge L (2021). High-valent bimetal Ni_3_S_2_/Co_3_S_4_ induced by Cu doping for bifunctional electrocatalytic water splitting. Appl. Catal. B Environ..

[17] Tang T, Jiang WJ, Niu S, Liu N, Luo H (2017). Electronic and morphological dual modulation of cobalt carbonate hydroxides by Mn doping toward highly efficient and stable bifunctional electrocatalysts for overall water splitting. J. Am. Chem. Soc..

[18] Wu T, Song E, Zhang S, Luo M, Zhao C (2021). Engineering metallic heterostructure based on Ni_3_N and 2M-MoS_2_ for alkaline water electrolysis with industry-compatible current density and stability. Adv. Mater..

[19] Wang P, Qi J, Li C, Chen X, Wang T (2020). N-doped carbon nanotubes encapsulating Ni/MoN heterostructures grown on carbon cloth for overall water splitting. ChemElectroChem.

[20] Guo Y, Yuan P, Zhang J, Xia H, Cheng F (2018). Co_2_P–CoN double active centers confined in N-doped carbon nanotube: heterostructural engineering for trifunctional catalysis toward HER, ORR, OER, and Zn–air batteries driven water splitting. Adv. Funct. Mater..

[21] Zhang L, Lu C, Ye F, Pang R, Liu Y (2021). Selenic acid etching assisted vacancy engineering for designing highly active electrocatalysts toward the oxygen evolution reaction. Adv. Mater..

[22] Duan J, Chen S, Ortiz-Ledon CA, Jaroniec M, Qiao SZ (2020). Phosphorus vacancies that boost electrocatalytic hydrogen evolution by two orders of magnitude. Angew. Chem. Int. Ed..

[23] Wang P, Zhu J, Pu Z, Qin R, Zhang C (2021). Interfacial engineering of Co nanoparticles/Co_2_C nanowires boosts overall water splitting kinetics. Appl. Catal. B Environ..

[24] Riyajuddin S, Azmi K, Pahuja M, Kumar S, Maruyama T (2021). Super-hydrophilic hierarchical Ni-foam-graphene-carbon nanotubes-Ni_2_P-CuP_2_ nano-architecture as efficient electrocatalyst for overall water splitting. ACS Nano.

[25] Zhang Q, Xiao W, Guo WH, Yang YX, Lei JL (2021). Macroporous array induced multiscale modulation at the surface/interface of Co(OH)_2_/NiMo self-supporting electrode for effective overall water splitting. Adv. Funct. Mater..

[26] Jin S (2017). Are metal chalcogenides, nitrides, and phosphides oxygen evolution catalysts or bifunctional catalysts?. ACS Energy Lett..

[27] Kornienko N, Heidary N, Cibin G, Reisner E (2018). Catalysis by design: development of a bifunctional water splitting catalyst through an operando measurement directed optimization cycle. Chem. Sci..

[28] Wang X, Li W, Xiong D, Petrovykh DY, Liu L (2016). Bifunctional nickel phosphide nanocatalysts supported on carbon fiber paper for highly efficient and stable overall water splitting. Adv. Funct. Mater..

[29] Li X, Han GQ, Liu YR, Dong B, Hu WH (2016). NiSe@NiOOH core–shell hyacinth-like nanostructures on nickel foam synthesized by in situ electrochemical oxidation as an efficient electrocatalyst for the oxygen evolution reaction. ACS Appl. Mater. Interfaces.

[30] Deng J, Deng D, Bao X (2017). Robust catalysis on 2D materials encapsulating metals: concept, application, and perspective. Adv. Mater..

[31] Wang P, Luo Y, Zhang G, Wu M, Chen Z (2022). MnO_x_-decorated nickel-iron phosphides nanosheets: interface modifications for robust overall water splitting at ultra-high current densities. Small.

[32] Deng J, Ren P, Deng D, Bao X (2015). Enhanced electron penetration through an ultrathin graphene layer for highly efficient catalysis of the hydrogen evolution reaction. Angew. Chem. Int. Ed..

[33] Wang P, Qi J, Li C, Li W, Wang T (2020). Hierarchical CoNi_2_S_4_@NiMn-layered double hydroxide heterostructure nanoarrays on superhydrophilic carbon cloth for enhanced overall water splitting. Electrochim. Acta.

[34] Hao Y, Li Y, Wu J, Meng L, Wang J (2021). Recognition of surface oxygen intermediates on NiFe oxyhydroxide oxygen-evolving catalysts by homogeneous oxidation reactivity. J. Am. Chem. Soc..

[35] Zhu K, Zhu X, Yang W (2018). Application of in situ techniques for the characterization of NiFe-based oxygen evolution reaction (OER) electrocatalysts. Angew. Chem. Int. Ed..

[36] Song F, Bai L, Moysiadou A, Lee S, Hu C (2018). Transition metal oxides as electrocatalysts for the oxygen evolution reaction in alkaline solutions: an application-inspired renaissance. J. Am. Chem. Soc..

[37] Zhang B, Li Y, Valvo M, Fan L, Daniel Q (2017). Electrocatalytic water oxidation promoted by 3D nano-architectured turbostratic δ-MnO_x_ on carbon nanotube. Chemsuschem.

[38] Long X, Chen Z, Ju M, Sun M, Jin L (2021). TM LDH meets birnessite: a 2D–2D hybrid catalyst with long-term stability for water oxidation at industrial operating conditions. Angew. Chem. Int. Ed..

[39] Chaudhari NK, Jin H, Kim B, Lee K (2017). Nanostructured materials on 3D nickel foam as electrocatalysts for water splitting. Nanoscale.

[40] Jiang N, Tang Q, Sheng M, You B, Jiang D (2016). Nickel sulfides for electrocatalytic hydrogen evolution under alkaline conditions: a case study of crystalline NiS, NiS_2_, and Ni_3_S_2_ nanoparticles. Catal. Sci. Technol..

[41] Zhang L, Zheng Y, Wang J, Geng Y, Zhang B (2021). Ni/Mo bimetallic-oxide-derived heterointerface-rich sulfide nanosheets with Co-doping for efficient alkaline hydrogen evolution by boosting volmer reaction. Small.

[42] Yuan J, Cheng X, Wang H, Lei C, Pardiwala S (2020). A superaerophobic bimetallic selenides heterostructure for efficient industrial-level oxygen evolution at ultra-high current densities. Nano-Micro Lett..

[43] Luo X, Ji P, Wang P, Cheng R, Chen D (2020). Interface engineering of hierarchical branched Mo-doped Ni_3_S_2_/Ni_x_P_y_ hollow heterostructure nanorods for efficient overall water splitting. Adv. Energy Mater..

[44] Li J, Xu W, Luo J, Zhou D, Zhang D (2017). Synthesis of 3D hexagram-like cobalt-manganese sulfides nanosheets grown on nickel foam: a bifunctional electrocatalyst for overall water splitting. Nano-Micro Lett..

[45] Suryawanshi MP, Ghorpade UV, Shin SW, Suryawanshi UP, Shim HJ (2018). Facile, room temperature, electroless deposited (Fe_1-x_, M_n_x)OOH nanosheets as advanced catalysts: the role of Mn incorporation. Small.

[46] Abe H, Murakami A, Tsunekawa S, Okada T, Wakabayashi T (2021). Selective catalyst for oxygen evolution in neutral brine electrolysis: an oxygen-deficient manganese oxide film. ACS Catal..

[47] Ravel B, Newville M (2005). Athena, artemis, hephaestus: data analysis for X-ray absorption spectroscopy using IFEFFIT. J. Synchrotron Rad..

[48] Hartmann F, Etter M, Cibin G, Liers L, Terraschke H (2021). Superior sodium storage properties in the anode material NiCr_2_S_4_ for sodium-ion batteries: an X-ray diffraction, pair distribution function, and X-ray absorption study reveals a conversion mechanism via nickel extrusion. Adv. Mater..

[49] Funke H, Scheinost AC, Chukalina M (2005). Wavelet analysis of extended X-ray absorption fine structure data. Phys. Rev. B.

[50] Wang Y, Yan L, Dastafkan K, Zhao C, Zhao X (2021). Lattice matching growth of conductive hierarchical porous MOF/LDH heteronanotube arrays for highly efficient water oxidation. Adv. Mater..

[51] Wang S, Yang P, Sun X, Xing H, Hu J (2021). Synthesis of 3D heterostructure Co-doped Fe_2_P electrocatalyst for overall seawater electrolysis. Appl. Catal. B Environ..

[52] Xu Z, Jin S, Seo MH, Wang X (2021). Hierarchical Ni-Mo_2_C/N-doped carbon mott-schottky array for water electrolysis. Appl. Catal. B Environ..

