# Supplementary material for: Interface Engineering of NixSy@MnOxHy Nanorods to Efficiently Enhance Overall-Water-Splitting Activity and Stability
Source: Nanomicro Lett. 2022 May 3;14:120. doi: 10.1007/s40820-022-00860-2 (PMC9065220; doi:10.1007/s40820-022-00860-2)
Supplement: Supplementary file 1 — Supplementary file1 (PDF 3005 kb) [file 40820_2022_860_MOESM1_ESM.pdf]

Supporting Information for

Interface Engineering of Ni_xS_y@MnO_xH_y Nanorods to Efficiently Enhance Overall-Water-Splitting Activity and Stability

Pan Wang[1, 2, 3], Yuanzhi Luo[1], Gaixia Zhang[3, *], Zhangsen Chen[3], Hariprasad Ranganathan[3], Shuhui Sun[3, *], and Zhicong Shi[1, *]

[1]Institute of Batteries, School of Materials and Energy, Guangdong University of Technology, Guangzhou 510006, P. R. China

[2]The Key Laboratory of Fuel Cell Technology of Guangdong Province, School of Chemistry and Chemical Engineering, South China University of Technology, Guangzhou 510641, P. R. China

[3]Énergie Matériaux Télécommunications Research Centre, Institut National de la Recherche Scientifique (INRS), Varennes, Québec, J3X 1P7, Canada

*Corresponding authors. E-mail: Gaixia.Zhang@inrs.ca (Gaixia Zhang); Shuhui@emt.inrs.ca (Shuhui Sun); zhicong@gdut.edu.cn (Zhicong Shi)

S1 Physical Characterization Detail of XAFS

The X-ray absorption fine spectroscopy (XAFS) measurements were performed at the Canadian Light Source (CLS) located at the University of Saskatchewan, a 2.9 GeV third-generation synchrotron source.

Hard X-ray Micro Analysis (HXMA) beamline: X-ray near-edge spectra (XANES) at Ni and *K*-edge were collected in total-fluorescence-yield mode using a 32-element Ge detector at ambient condition on the 06ID-1 Hard X-ray Micro Analysis (HXMA) beamline at the Canadian Light Source (CLS). During the data collection, the CLS storage ring (2.9 GeV) was operated under 250 mA mode and the HXMA superconducting wiggler was run at 1.9 T. The scan range was kept in an energy range of 8127–9292 eV for Ni *K*-edge and 6557–7278 eV for Mn *K*-edge. Data collection configuration was using metal Ni and Mn foils as energy calibration by in step calibration for every data set. The baseline of pre-edge was subtracted and the post-edge was normalized in the spectra.

Soft X-ray micro characterization beamline (SXRMB): The S *K*-edge X-ray absorption near-edge structure (XANES) and Extended X-ray Absorption Fine Structure (EXAFS) data were collected on the soft X-ray micro characterization beamline (SXRMB) beamline. The SXRMB beamline used a Si(111) double crystal monochromator to cover the 2–10 keV energy range with a resolving power of 10 000. The XAS measurements were performed in fluorescence mode using a 4-element Si(Li) drift detector in a vacuum chamber. Analyses of both the near edge (on an energy scale) and extended range (in the R space) XAS spectra were performed using Athena software.

Spherical Grating Monochromator (SGM): The XANES at the Mn *L*-edge were obtained at the spherical grating monochromator (SGM) beamline with an energy resolution of $E/\Delta E \geq 5000$. The spectra were recorded in partial X-ray fluorescence yield (PFY) mode using four silicon drift detectors (SDD) under 10–6 Torr with a beam spot size of 25 μm. Data were first normalized to the incident photon flux I0 measured with a refreshed gold mesh at SGM before the measurement.

S2 Supplementary Figures and Tables

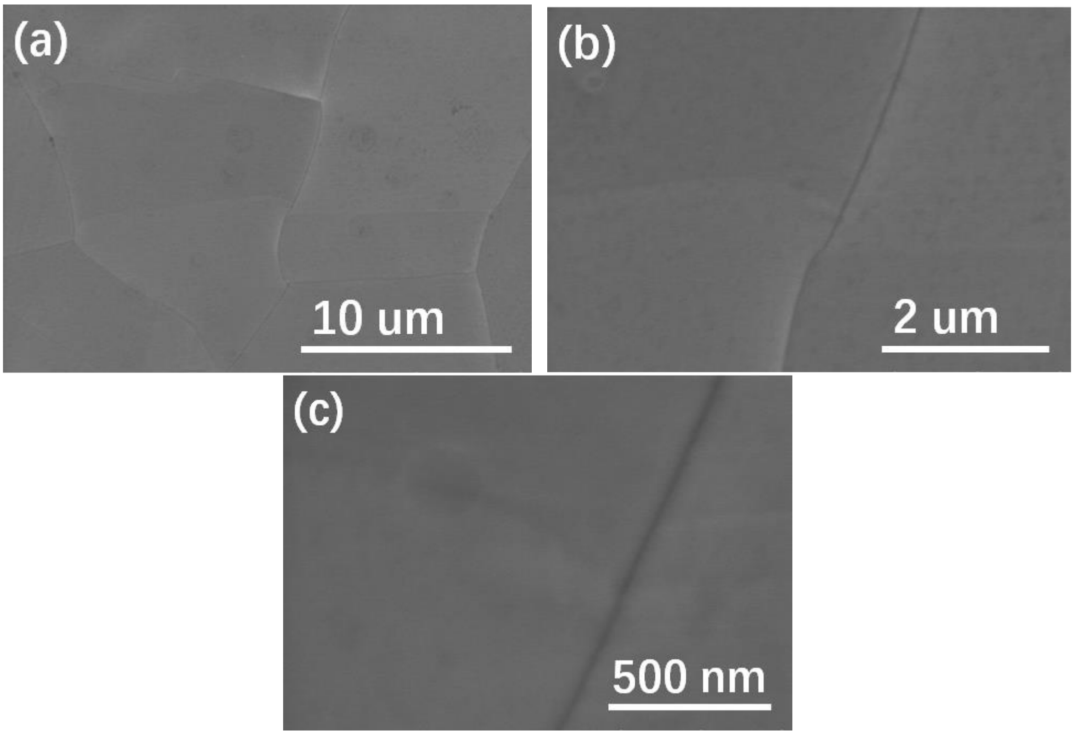

Fig. S1 (**a**–**c**) SEM images of pure NF

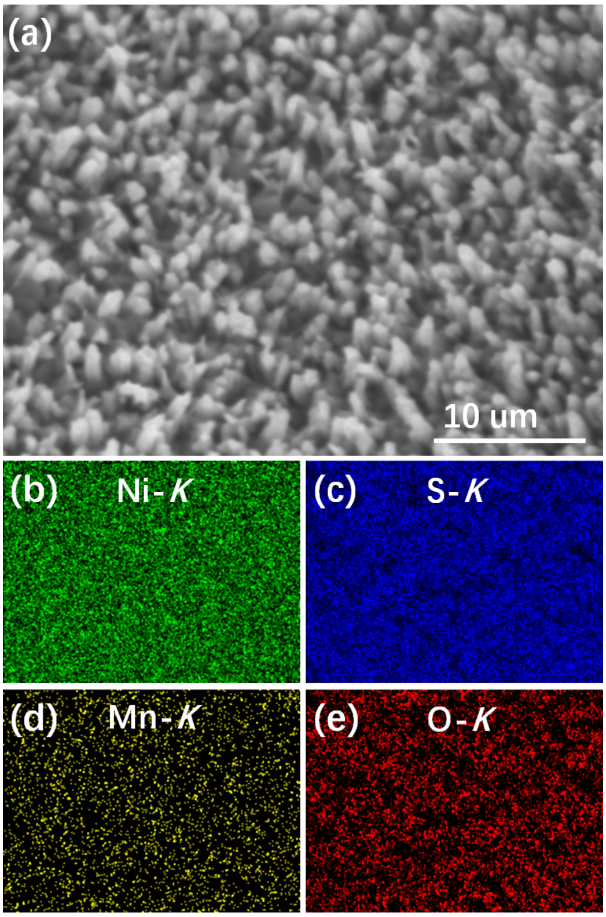

Fig. S2 SEM image and the corresponding elemental mapping images of $Ni_xS_y@MnO_xH_y/NF$

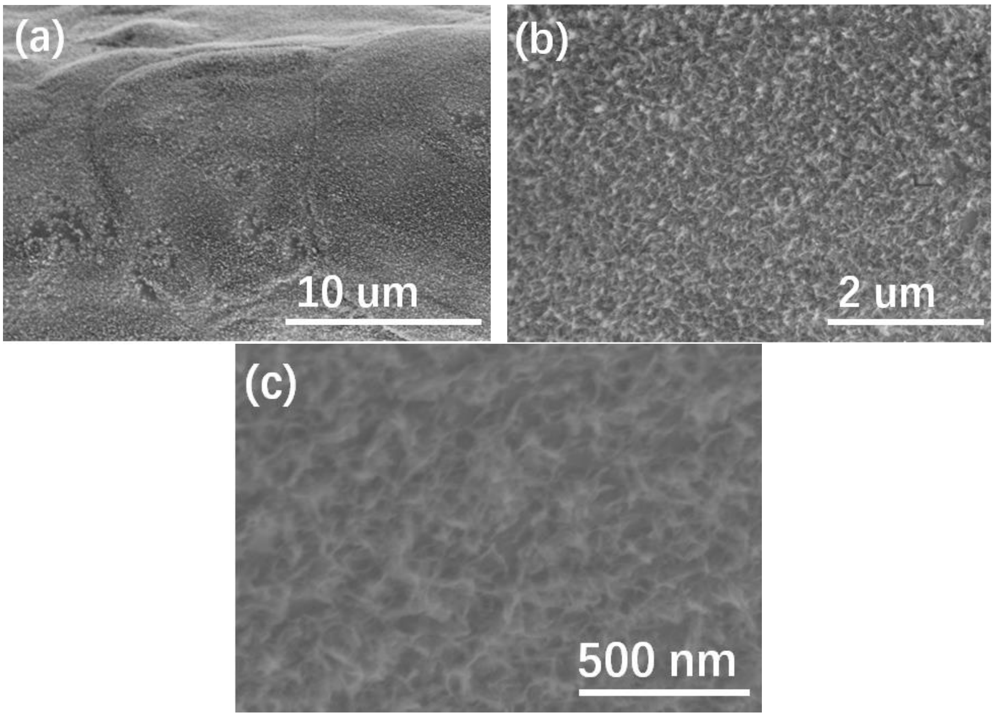

Fig. S3 (**a**–**c**) SEM images of MnO_xH_y/NF

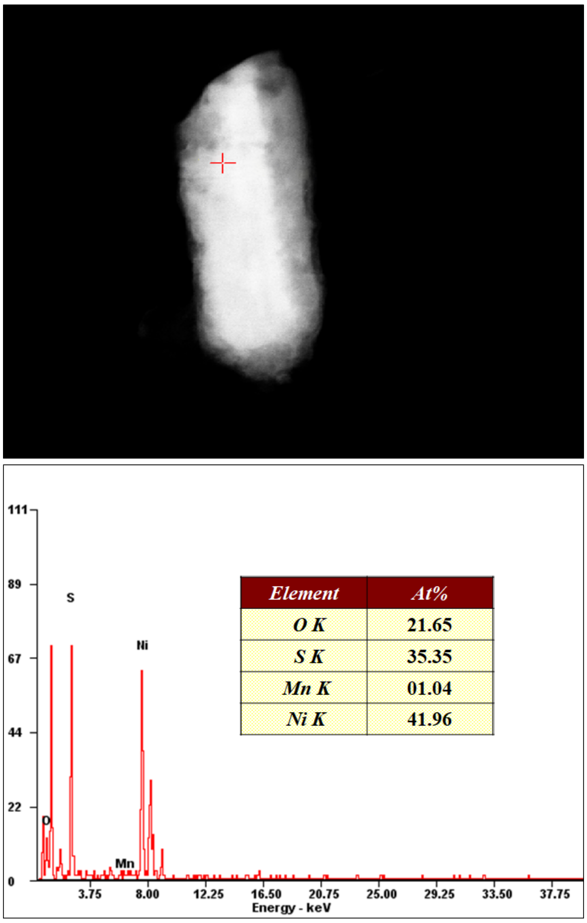

Fig. S4 STEM image of Ni_xS_y@MnO_xH_y nanorod and its corresponding EDX spectrum

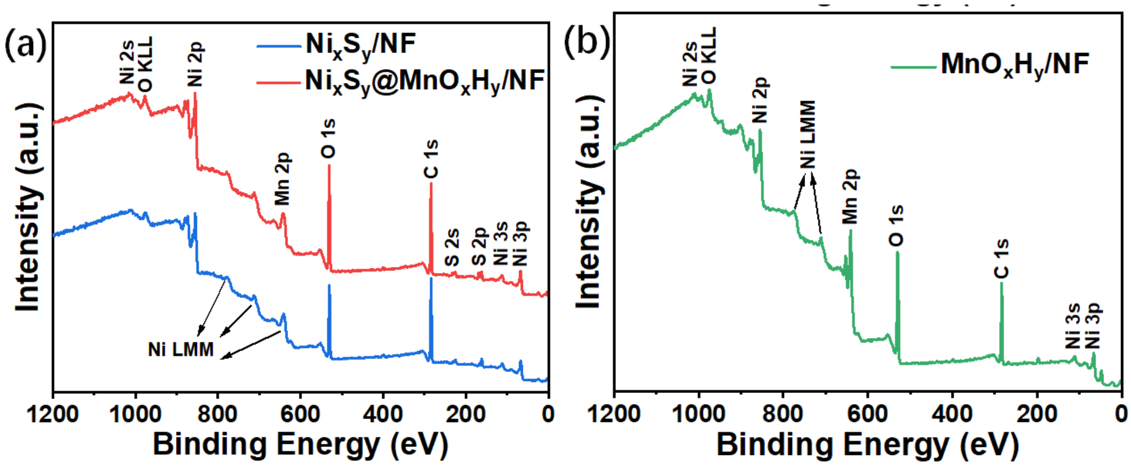

Fig. S5 XPS survey spectra of (**a**) Ni_xS_y/NF, Ni_xS_y@MnO_xH_y/NF, and (**b**) MnO_xH_y/NF

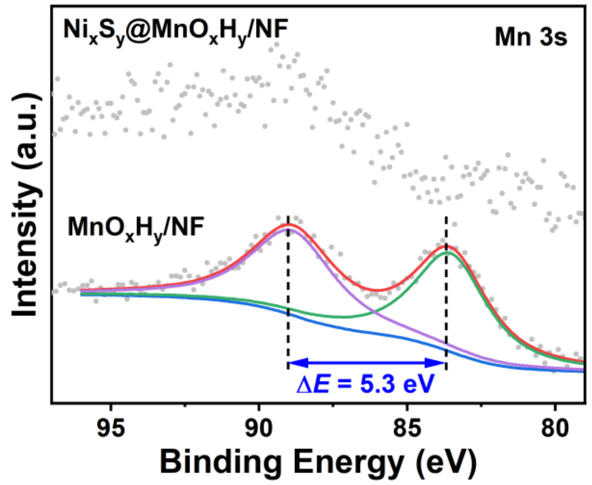

Fig. S6 High-resolution XPS spectra of Mn 3s for MnO_xH_y/NF and Ni_xS_y@MnO_xH_y/NF

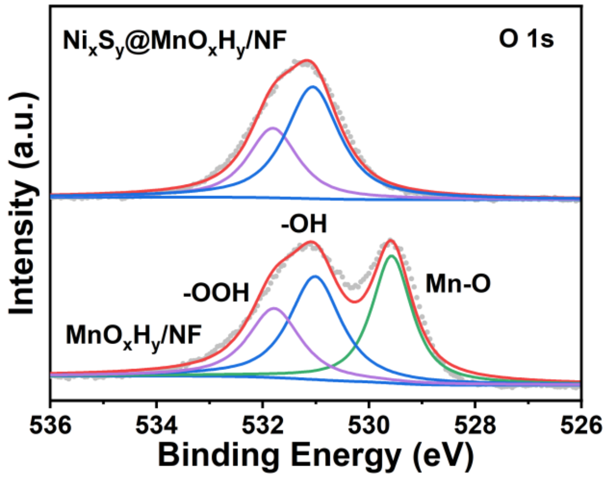

Fig. S7 High-resolution XPS spectra of O 1s for MnO_xH_y/NF and Ni_xS_y@MnO_xH_y/NF

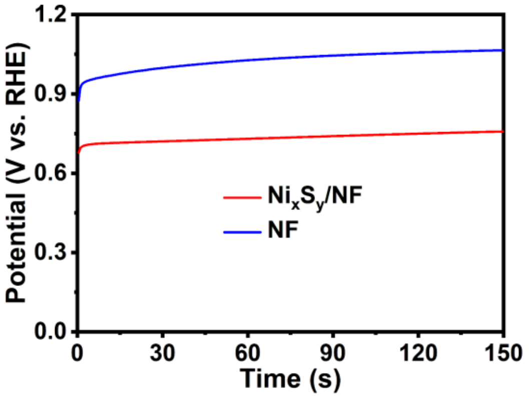

Fig. S8 Chronopotentiometry curves of Ni_xS_y/NF and NF at 0.5 mA cm^{-2} for 150 s during the electrodeposition process

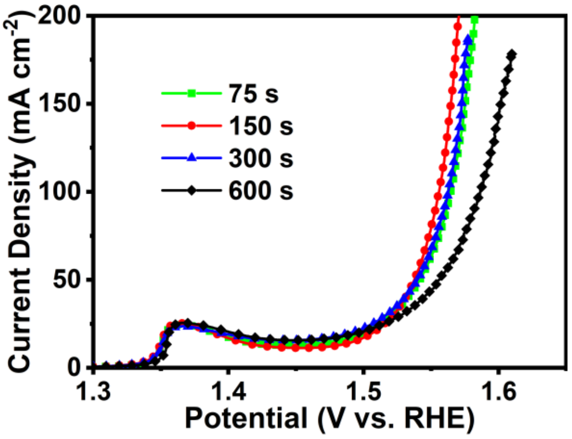

Fig. S9 LSV curves of Ni_xS_y@MnO_xH_y/NF with different electrodeposition time for OER

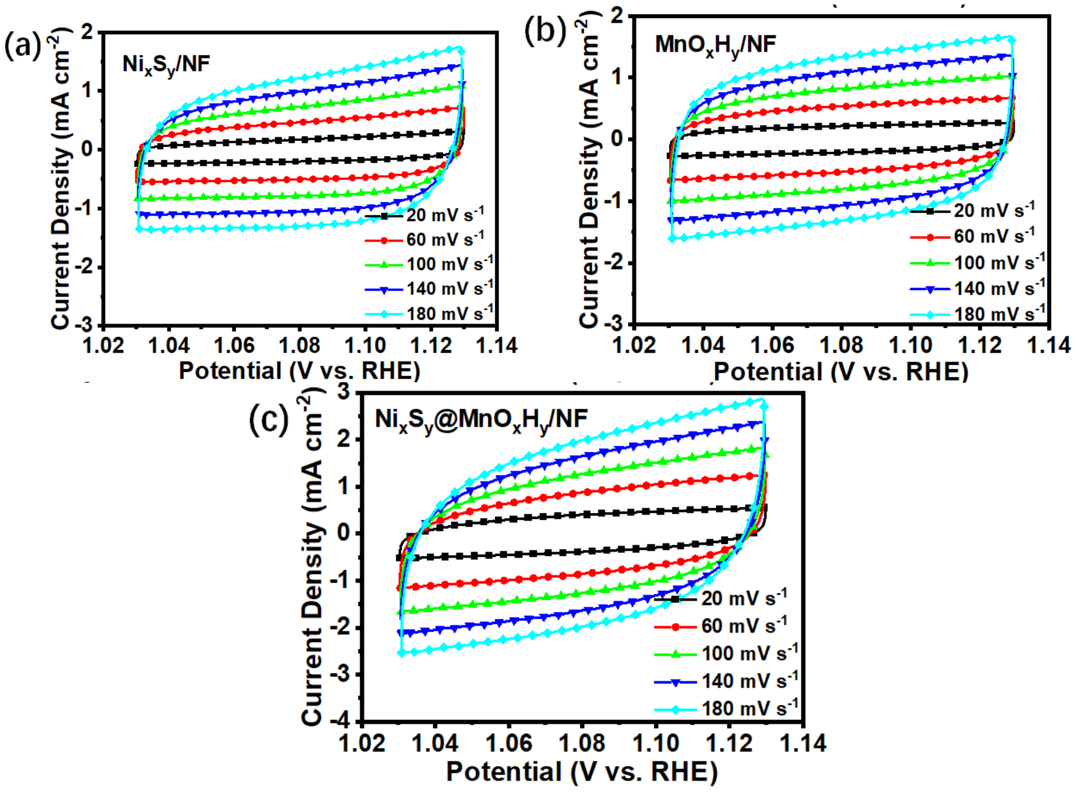

Fig. S10 CV curves in the region of 1.03–1.13 V for (**a**) Ni_xS_y/NF, (**b**) MnO_xH_y/NF, and (**c**) Ni_xS_y@MnO_xH_y/NF at various scan rates (20, 60, 100 mV s^{-1} etc.)

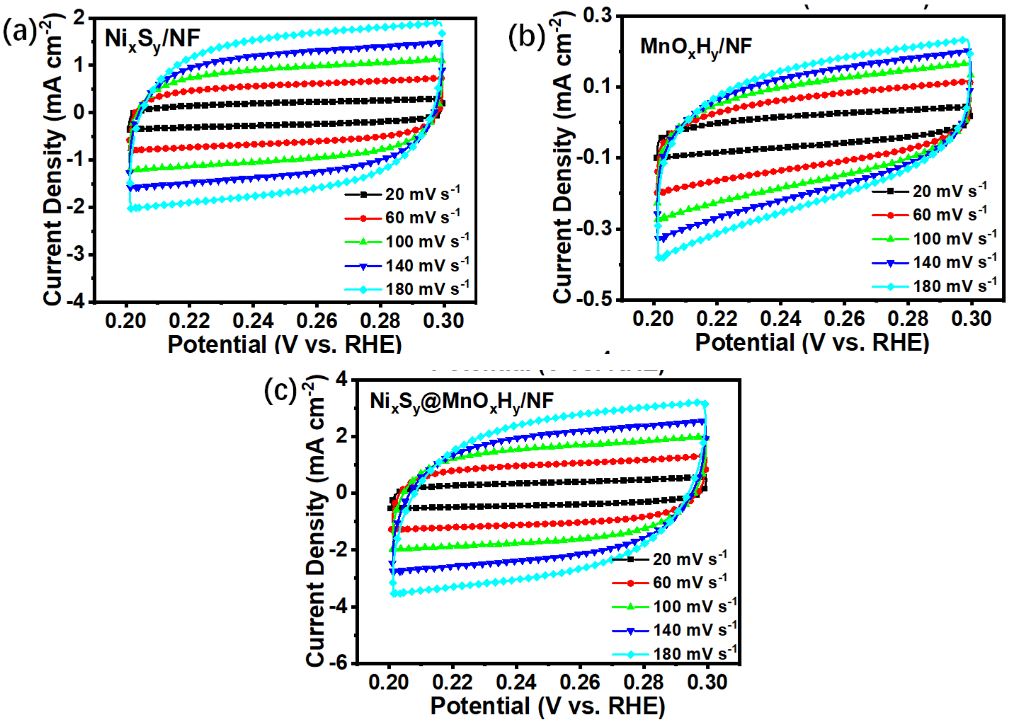

Fig. S11 CV curves in the region of 0.2–0.3 V for (**a**) Ni_xS_y/NF, (**b**) MnO_xH_y/NF, and (**c**) Ni_xS_y@MnO_xH_y/NF at various scan rates (20, 60, 100 mV s^{-1} etc.)

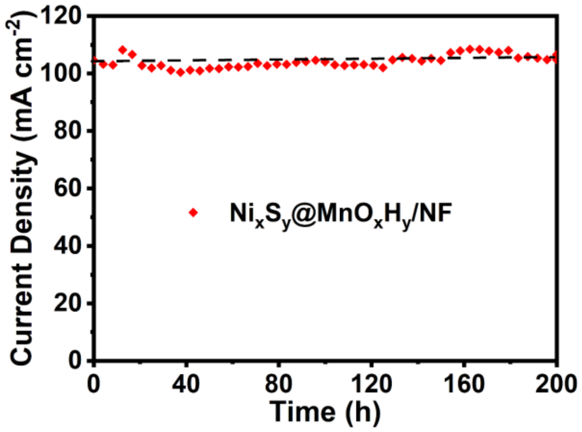

Fig. S12 Chronoamperometry curve of Ni_xS_y@MnO_xH_y/NF as both the anode and cathode for overall water splitting

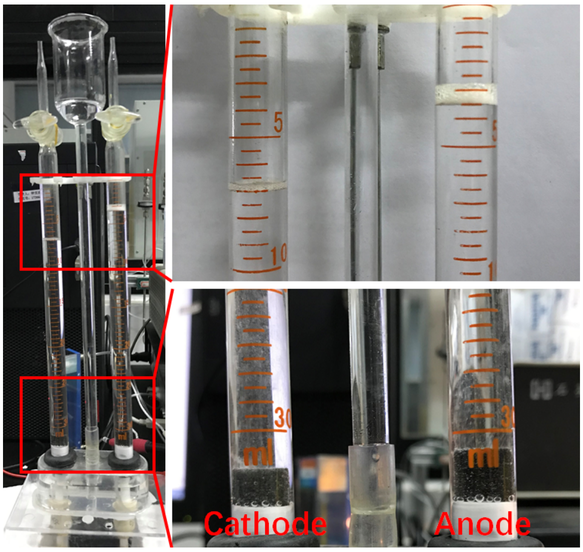

Fig. S13 The picture of the drainage method

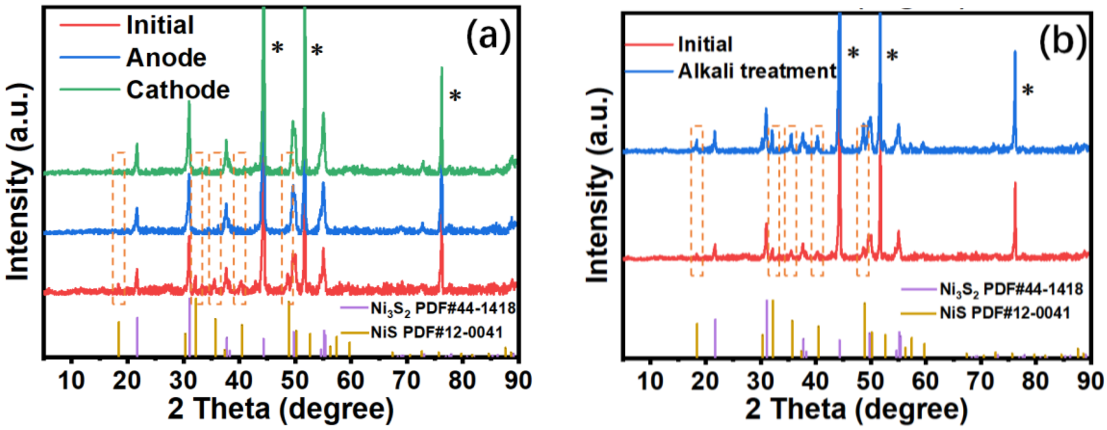

Fig. S14 (**a**) XRD patterns of initial $Ni_xS_y@MnO_xH_y/NF$ and the corresponding anode and cathode after the stability test. (**b**) XRD patterns of initial $Ni_xS_y@MnO_xH_y/NF$ and the corresponding $Ni_xS_y@MnO_xH_y/NF$ after alkali treatment for 100 h

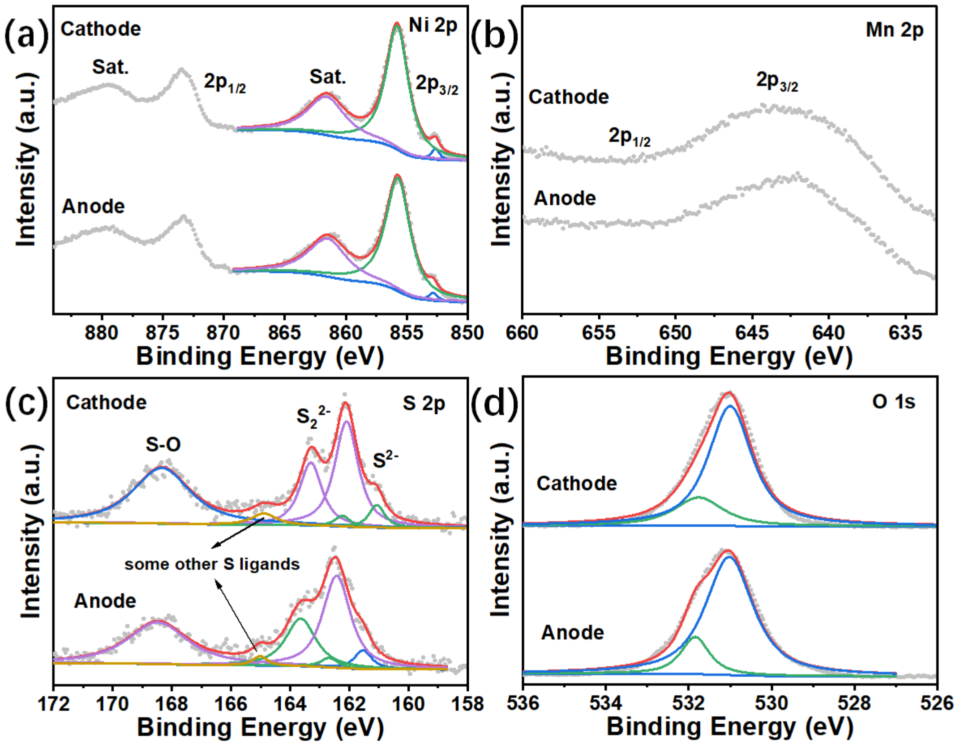

Fig. S15 High-resolution XPS spectra of (**a**) Ni 2p, (**b**) Mn 2p, (**c**) S 2p, and (**d**) O 1s for the cathode and anode after the stability test

Table S1 Comparison of overpotentials at 100 and 500 mA cm^{-2} and stabilities of recently different non-noble metal OER electrocatalysts in 1.0 M KOH solution

**Electrocatalyst**	**Overpotential (mV) at 100 mA cm^{-2}**	**Overpotential (mV) at 500 mA cm^{-2}**	**Stability (t/h@j/mA cm^{-2})**	**Refs.**
$Ni_xS_y@MnO_xH_y$/NF	326	356	150 h@100 mA cm^{-2}	This work
Ni_xS_y/NF	381	-	Bad	This work
GDs/$Co_{0.8}Ni_{0.2}P$ @Cu nanowires	350	-	50 h@50 mA cm^{-2}	*ACS Appl. Mater. Interfaces*, **2017**, 9, 24600
NG-NiFe@MoC_2	400	-	-	*Nano Energy*, **2018**, 50, 212
$Co(S_xSe_{1-x})_2$/carbon fibers	370	-	20 h@10 mA cm^{-2}	*Adv. Funct. Mater.*, **2017**, 27, 1701008
$NiCo_2S_4$/NF	370	-	10 h@10 mA cm^{-2}	*Adv. Funct. Mater.*, **2016**, 26, 4661
Mo-doped CoP/carbon cloth	360	-	20 h@45 mA cm^{-2}	*Nano Energy*, **2018**, 48, 73
$NiS_2/NiSe_2$	400	-	20 h@100 mA cm^{-2}	*Small*, **2020**, 16, 1905083.
NiCoP@NiMn LDH/NF	293	320	100 h@50 mA cm^{-2}	*ACS Appl. Mater. Interfaces*, **2020**, 12, 4385
$Fe_2O_3@Ni_2P/Ni(PO_3)_2$/NF	300	340	8 h@200 mA cm^{-2}	*J. Mater. Chem. A*, **2019**, 7, 965
$Ni_xCo_{3-x}S_4/Ni_3S_2$/Ni Foam	330	480	30 h@10 mA cm^{-2}	*Nano Energy*, ***2017***, *35, 161*

$Co_{0.85}Se_{1-x}$@C nanocages	320	-	50 h@20 mA cm^{-2}	*Adv. Mater.*, **2021**, 33, 2007523
$NiCo_{2-x}Fe_xO_4$ Nanoboxes	330	-	25 h@50 mA cm^{-2}	*Angew. Chem. Int. Ed.*, **2021**, 60, 11841
Fe-doped Ni_3C/NC	350	-	10 h@10 mA cm^{-2}	*Angew. Chem. Int. Ed.*, **2017**, 129, 12740
Ni-Co-Se nanocages	400	-	20 h@100 mA cm^{-2}	*ACS Sustainable Chem. Eng.*, **2018**, *6*, 10952
CeO_x/CoO_x	420	-	15 h@15 mA cm^{-2}	*ACS Catal.* **2018**, *8*, 4257
NiS/Ni-BDC	440	-	12 h@20 mA cm^{-2}	*ACS Appl. Mater. Interfaces*, **2019**, *11*, 41595
CoP/TiO_x	410	-	8.3 h@10 mA cm^{-2}	*Small*, **2019**, *16*, 1905075

Table S2 Comparison of overpotentials at 10 and 100 mA cm^{-2} and stabilities of recently different non-noble metal HER electrocatalysts in 1.0 M KOH solution

**Electrocatalyst**	**Overpotential (mV) at 10 mA cm^{-2}**	**Overpotential (mV) at 100 mA cm^{-2}**	**Stability (t/h@j/mA cm^{-2})**	**Refs.**
$Ni_xS_y@MnO_xH_y$/NF	179	270	100 h@100 mA cm^{-2}	This work
Ni_xS_y/NF	193	295	-	This work
VOOH/NF	164	270	24 h@10 mA cm^{-2}	*Angew. Chem. Int. Ed.*, **2017**, 56, 573
HC-MoS_2/Mo_2C	-	330	-	*Nat. Commun.*, **2020**, 11, 3724
$Fe_{0.95-x}Ni_xS_{1.05}$ nanosheets	263	-	-	*J. Mater. Chem. A*, **2020**, 8, 20323
$NiO/NiFe_2O_4$	282	-	-	*Small*, **2021**, 17, 2103501.
N-MoO_2/Ni_3S_2/NF	-	300	10 h@500 mA cm^{-2}	*ACS Appl. Mater. Interfaces*, **2019**, 11, 27743
$Co_2P/Mo_3Co_3C/Mo_2C$@C	182	-	-	*J. Mater. Chem. A*, **2018**, 6, 5789
Ni-GF/VC	128	240	20 h@10 mA cm^{-2}	*Adv. Energy Mater.*, **2020**, 10, 2002260
Ni single atom/NC	102	255	14 h@50 mA cm^{-2}	*Adv. Mater.*, **2021**, 33, 2003846
Co/Mo_2C@NC	218	300	14 h@10 mA cm^{-2}	*J. Mater. Chem. A*, **2017**, *5, 16929*
NG-NiFe@MoC_2	150	290	-	*Nano Energy*, **2018**, *50, 212*
NiO nanorod arrays	110	280	10 h@10 mA cm^{-2}	*Nano Energy*, **2018**, 43, 103

Co_4Mo_2@NC	218	300	14 h@10 mA cm^{-2}	*J. Mater. Chem. A*, **2017**, *5*, 16929
Ni/Mo_2C-PC	179	-	10 h@10 mA cm^{-2}	*Chem. Sci.*, **2017**, 8, 968
Co/porous N-rich carbon	298	470	10 h@10 mA cm^{-2}	*J. Mater. Chem. A*, **2016**, 4, 3204
Ni_3S_2 nanowires	199.2	-	30 h@20 mA cm^{-2}	*Int. J. Hydrogen Energy*, **2017**, 42, 7136
$Co_{0.75}Fe_{0.25}$-NC	202	-	45 h@10 mA cm^{-2}	*J. Power Sources*, **2018**, 389, 249

Table S3 Comparison of cell voltages at 10 and 100 mA cm^{-2} and stabilities of recently different bifunctional non-noble metal electrocatalysts for overall water splitting in 1.0 M KOH solution

**Electrocatalyst**	**Cell voltage at 10 mA cm^{-2} (V)**	**Cell voltage at 100 mA cm^{-2} (V)**	**Stability (t/h@j/mA cm^{-2})**	**Refs.**
Ni_xS_y@MnO_xH_y/NF	1.529	1.828	100 h@100 mA cm^{-2}	This work
Co-Co_2C/CC	1.63	1.98	20 h@20 mA cm^{-2}	*Appl. Catal. B: Environ.*, **2021**, 296, 120334
CoP-InNC@CNT	1.58	1.86	15 h@10 mA cm^{-2}	*Adv. Sci.*, **2020**, 7, 1903195.
Ni_3FeN/r-GO	1.6	1.96	100 h@10 mA cm^{-2}	*ACS Nano*, **2018**, 12, 245
FeCoNi/CC	1.55	2.00	12 h@100 mA cm^{-2}	*Adv. Energy Mater.*, **2019**, 9, 1901312
Co_3S_4/MOF	1.55	1.90	24 h@10 mA cm^{-2}	*Adv. Mater.*, **2019**, 31, 1806672
VOOH/NF	1.62	1.83	50 h@50 mA cm^{-2}	*Angew. Chem. Int. Ed.*, **2017**, 56, 573
NiFe-MOFs/NF	1.495	1.647	100 h@100 mA cm^{-2}	*Nano Energy*, **2019**, 57, 1
Cu@NiFe LDH/Cu foam	1.54	1.69	24 h@100 mA cm^{-2}	*Energy Environ. Sci.*, **2017**, 10, 1820
NC@$CuCo_2N_x$/ Carbon fiber	1.62	1.86	60 h@10 mA cm^{-2}	*Adv. Funct. Mater.*, **2017**, 27, 1704169
